# Metformin Sensitizes PTEN-deficient Prostate Cancer to PARP Inhibitors by Rebuilding NADP^+^ Homeostasis

**DOI:** 10.7150/ijbs.121033

**Published:** 2026-01-08

**Authors:** Xiaodong Hao, Zheng Chao, Hao Peng, Xiangdong Guo, Shuo Zheng, Chunyu Zhang, Hao Ding, Yanan Wang, Zirui Xi, Yuan Gao, Guanyu Qu, Yao Zhu, Zhiqiang Chen, Peixiang Lan, Le Li, Zhihua Wang

**Affiliations:** 1 Department of Urology, Tongji Hospital, Tongji Medical College, Huazhong University of Science and Technology, Wuhan, China.; 2 Institute of Organ Transplantation, Tongji Hospital, Tongji Medical College, Huazhong University of Science and Technology, Key Laboratory of Organ Transplantation, Ministry of Education, NHC Key Laboratory of Organ Transplantation, Key Laboratory of Organ Transplantation, Chinese Academy of Medical Sciences, Wuhan, China.; 3 Department of Urology, The First Hospital of Hebei Medical University, Shijiazhuang, China.; 4 Department of Urology, Liaocheng People s Hospital, Liaocheng, China.; 5 Department of Urology, Fudan University Affiliated Cancer Hospital, Shanghai, China.

**Keywords:** PARP inhibitor, PTEN-deficient PCa, metformin, machine learning model, NADP^+^

## Abstract

**Purpose:** DNA repair and DNA damage responses in cancer cells are regulated by metabolic reprogramming, which is increasingly recognized as a key factor contributing to PARP inhibitor (PARPi) treatment failure. This study aims to explore the metabolic mechanisms underlying PARPi resistance in PTEN-deficient prostate cancer and identify clinically viable metabolic interventions to overcome therapy failure.

**Experimental Design:** A multicenter retrospective cohort was analyzed to evaluate the efficacy of combined metformin-PARPi therapy. Mechanistic studies utilized molecular assays to elucidate PARPi resistance and its critical determinants. Machine learning models predicting PARPi response were developed using clinical datasets and interpreted via SHAP analysis.

**Results:** In PTEN-deficient cancer cells, lactate accumulation activated the NHE1/PKC/NOX1 axis, sustaining elevated NADP^+^ levels. NADP^+^ competitively inhibited the formation of PARPi-PARP-DNA complexes, leading to PARPi resistance. However, metformin administration significantly elevated NADP^+^ levels, inducing allosteric effects on PARP structures and enhancing PARPi efficacy. Based on these findings, we developed and validated a predictive machine learning model for PARPi response, which was interpreted using SHAP and deployed on a web platform.

**Conclusions:** Metformin modulates NADP^+^ levels to influence PARPi sensitivity in PTEN-deficient prostate cancer. Additionally, we developed a machine learning model to provide clinicians with personalized predictions for PARPi response.

## Introduction

Prostate cancer (PCa) is one of the most significant threats to men's health. Among Western men, its incidence is the highest among all malignancies, while its mortality rate ranks second 1. Since patients progressing to castration-resistant prostate cancer (CRPC) typically have poor outcomes 2, developing effective CRPC therapies has long been a research priority. The PROfound study confirmed that in metastatic CRPC (mCRPC) patients with homologous recombination repair (HRR) gene mutations, PARP inhibitors (PARPi) demonstrated significant therapeutic benefits with an acceptable safety profile 3. For CRPC patients with BRCA1/2 and ATM mutations, olaparib significantly prolonged radiographic progression-free survival (rPFS) and overall survival (OS) 4. However, clinical data indicate that the proportion of patients carrying BRCA1/2 and other gene mutations is relatively low 5, limiting the clinical application of PARPi therapy 6.

Notably, PARPi have also demonstrated efficacy in other cancer types lacking BRCA1/2 mutations or homologous recombination (HR) deficiency 7, 8. Additionally, PARPi combination therapies have shown superior efficacy to monotherapy in multiple clinical trials 9-12. This suggests additional molecules beyond BRCA mutations modulate PARPi sensitivity. PTEN loss is observed in ~50% of mCRPC cases 13-15. Given that nuclear PTEN plays a critical role in regulating DNA repair and cellular response to genotoxic stress 16, researchers hypothesized that PTEN-deficient prostate cancer might exhibit enhanced susceptibility to PARPi 17. However, clinical trials showed PARPi monotherapy failed to improve survival in PTEN-deficient PCa 18.

Studies have indicated that the accumulation of metabolic waste products such as lactate in the PTEN-deficient PCa microenvironment may be a key factor reducing the sensitivity of PTEN-deficient cancer cells to PARPi therapy 19. On one hand, the profound metabolic reprogramming of tumor cells can directly affect their DNA damage repair (DDR) capacity by altering energy supply patterns 20-22. On the other hand, specific metabolic intermediates such as NADP^+^, ATP, and NADPH are believed to participate in DNA damage responses through competitive inhibition 23. Despite these findings, the exact mechanisms of lactate-mediated PARPi resistance in PTEN-deficient PCa require further investigation. More importantly, current evidence remains insufficient to determine which combination therapy could effectively improve survival in PTEN-deficient PCa patients.

In this study, we initially discovered—based on multicenter follow-up cohort results—that metformin (Met) effectively enhanced PARPi efficacy in PTEN-deficient PCa patients. Subsequently, we identified that the tumor microenvironment of PTEN-deficient PCa exhibits moderately elevated levels of NADP⁺. This elevated NADP^+^ competitively inhibits PARPi-PARP-DNA complex formation, consequently reducing PARPi cytotoxicity. Furthermore, Met modulates NADP^+^ levels to potentiate PARPi's therapeutic effect. Building upon these mechanistic insights, we developed a machine learning model to predict PARPi response, providing clinicians with predictive tools for personalized prostate cancer treatment.

## Materials and Methods

### Patients and treatment

This study received ethical approval from the institutional review boards of all participating centers. We conducted a retrospective analysis of mCRPC patients treated with olaparib between 2017 and 2024 at four tertiary referral centers in China. The study included 461 CRPC patients who had genetically confirmed DDR gene defects. All patients had experienced disease progression after treatment with first- or second-line therapy for CRPC. Patients initially received olaparib 300 mg twice daily, with dose reduction to 450 mg once daily permitted for grade 3 adverse events (AEs).

### Clinical outcomes and treatment safety

Following olaparib treatment, patients underwent routine follow-up every 2-4 weeks to monitor: Symptom changes, Physical condition, Drug tolerance, and Laboratory parameters (complete blood count, liver and kidney function, electrolytes, and prostate-specific antigen [PSA]).

Patients underwent imaging assessments every 2-3 months until disease progression or intolerable toxicity occurred. Follow-up frequency was adjusted dynamically based on individual patient conditions. Progression-free survival (PFS) was defined as the time from treatment initiation to any of the following events: PSA level increase, Radiologic progression per Response Evaluation Criteria in Solid Tumors (RECIST 1.1) or Prostate Cancer Clinical Trials Working Group (PCWG3) criteria, Clinical symptom progression, Death. OS referred to the time from treatment initiation to death from any cause. PSA response was defined as: Any decline in PSA level from baseline (any PSA response) 24. The primary efficacy endpoint was PSA50 response (≥50% reduction in PSA from baseline) after PARPi treatment. Secondary endpoints included PFS and OS. Treatment safety was assessed using the Common Terminology Criteria for Adverse Events version 5.0 (CTCAE v5.0), with adverse event (AE) severity graded and recorded.

### Model development and comparison

Data from 461 patients treated at Tongji Medical Center were used to develop the prediction model, with the dataset split into training and validation sets at an 8:2 ratio. A total of 11 predictive factors were selected during the modeling process. We compared 11 machine learning algorithms (AdaBoost, ANN, DT, ET, GBM, KNN, LightGBM, LR, RF, SVM, and XGBoost) for predicting PSA50 response in patients after PARPi treatment. We performed hyperparameter optimization through 5-fold cross-validation with grid search to identify optimal hyperparameters by evaluating multiple parameter combinations. Model performance was assessed using metrics such as the Area Under the Receiver Operating Characteristic Curve (ROC), sensitivity, specificity, Positive Predictive Value (PPV), Negative Predictive Value (NPV), accuracy, and F1 score.

### Feature selection and model interpretation

Interpreting machine learning model predictions presents significant challenges. The SHAP (SHapley Additive exPlanations) method offers a robust solution to the "black box" problem of model interpretability, enabling both feature importance ranking and prediction outcome explanation. In this study, we analyzed the top-performing model identified during feature selection. SHAP provides dual interpretation perspectives: (1) global explanations quantifying feature contributions and revealing associations between inputs and PSA50 response, and (2) local explanations generating patient-specific interpretations from particular data inputs.

### Web-based deployment tool using the Streamlit framework

To facilitate the practical application of this model in clinical settings, we deployed the final prediction model as a web application using the Streamlit Python framework. When users input the relevant feature values required by the final model, the application outputs the probability of PSA50 response (50% reduction in prostate-specific antigen levels) and provides intuitive visualizations along with LIME (Local Interpretable Model-agnostic Explanations) interpretations for individual patients.

### Cell culture

In this study, four prostate cancer cell lines—PC3, DU145, 22Rv1, and LNCaP—were selected, all obtained from Wuhan Pricella Biotechnology Co., Ltd. (Wuhan, China). Among them, PC3 and LNCaP were PTEN-deficient, whereas DU145 and 22Rv1 were PTEN-positive. All cell cultures were supplemented with 100 μg/mL penicillin-streptomycin (Gibco) and maintained in Gibco's RPMI 1640 medium supplemented with 10% heat-inactivated FBS (Gemini) at 37 °C with 5% CO₂.

### CCK8 assay

We evaluated drug sensitivity in prostate cancer cells using the CCK8 assay. First, prostate cancer cells were seeded at a density of 2,000 cells/well in flat-bottom 96-well plates, with a final volume of 200 μL per well. After 48 hours of treatment with drugs of varying concentrations, 10 μL of CCK8 reagent was added to each well, followed by gentle shaking to ensure homogeneous distribution. The plates were incubated at 37 °C for 0.5-4 hours. Absorbance at 450 nm was measured using a microplate reader. Each condition was tested in triplicate, and standard deviations were calculated. We calculated IC50 values using nonlinear regression analysis in GraphPad Prism 7.

### Colony formation assay

We seeded PCa cells in plates and initiated drug treatment 24 hours later. We refreshed the drug-containing medium every three days. At the endpoint, cells were washed with phosphate-buffered saline (PBS), fixed, and stained with a 5% crystal violet solution. Images of the stained colonies were captured using a molecular imager (Bio-Rad Labs, Hercules, CA, USA). The number of distinct colonies was quantified using ImageJ software.

### Comet assay

After incubating prostate cancer cells in drug-containing medium for 12 hours, they were embedded on agarose gel-coated slides. The slides were then treated with neutral lysis solution (containing 2% Sarkosyl, 0.5 M EDTA, 0.5 mg/mL proteinase K, pH 8.0) for 1 hour at room temperature. We performed electrophoresis at 15-20 V (0.6 V/cm field strength) for 25 minutes, followed by staining with propidium iodide. Fluorescence microscopy was used to image the stained slides, and DNA migration rates in comet tails were analyzed using CometScore software.

### Flow cytometry for apoptosis

PCa cells were seeded at a density of 2 × 10⁵ cells/well in 6-well plates. After 24 hours, they were treated with various drug concentrations for 48 hours. We collected the treated cells, stained them with Annexin V-FITC and PI, and performed flow cytometry analysis, generating bivariate scatter plots with FITC fluorescence (Annexin V) on the x-axis and PI fluorescence on the y-axis.

### 3D tumor spheroid culture

Three-dimensional spheroid culture experiments were conducted as previously described 25. PCa cells (500 cells/well) were seeded in ultra-low attachment U-bottom 96-well plates (200 μL final volume). After 72 hours in complete medium (10% FBS), we replaced the medium with drug-containing solutions. Following 72 hours of drug treatment, live and dead cells were dual-stained with Calcein AM and propidium iodide. The 3D cultures were imaged using an inverted phase-contrast microscope (Leica Microsystems GmbH, Wetzlar, Germany). Fluorescence intensity was quantitatively analyzed using ImageJ software.

### Immunofluorescence

Immunofluorescence staining was performed according to standard protocols 26. Primary antibodies against PTEN and p-NOX1, along with fluorescently labeled secondary antibodies, were employed. Fluorescent images were captured using the Olympus inverted microscope.

### Western blot

Cells were lysed and precipitated using ice-cold RIPA buffer supplemented with a cocktail of protease and phosphatase inhibitors (Thermo Fisher). Protein concentrations were determined using the DC Protein Assay (Bio-Rad). Equal amounts of protein extracts (40-60 μg) were separated by SDS-PAGE and subsequently transferred to polyvinylidene fluoride (PVDF) membranes. The membranes were blocked with 5% non-fat dry milk in TBS containing 0.05% Tween 20 (Bio-Rad) for 45 minutes at room temperature, followed by overnight incubation at 4 °C with primary antibodies, and then incubated with anti-mouse IgG fluorescently labeled (1:2000, Rockland Immunochemical, #RL610-145-002) or anti-rabbit IgG (1:2000, Molecular Probes, #A-21109) for 1 hour at room temperature. Protein blots were visualized using an Odyssey scanner (LI-COR). Uncropped blots are included in the source data file. Detailed information regarding primary antibodies is as follows: PTEN polyclonal antibody, #A11193, 1:1000, Proteintech; NHE1 polyclonal antibody, #29761-1-AP, 1:1000, Proteintech; NOX1 antibody, #DF8684, 1:1000, Affinity; Phospho-NCF1/p47-phox (Ser359) antibody, #AF3167, 1:1000, Affinity; β-actin rabbit antibody (#AC038), 1:1000, Abclonall.

### Measurement of NAD⁺ and NADP⁺ concentrations

We resuspended 1 × 10^6 cells in 100 μL of NADP⁺ extraction buffer. We heated the cell lysates at 60 °C for 5 minutes and then added 20 μL of either NAD⁺ or NADP⁺ assay buffer. We quantified NAD⁺ and NADP⁺ levels through an enzymatic cycling assay that sequentially uses LDH and G6PDH. Absorbance at 565 nm was recorded using a microplate reader (Bio-Tek Instruments, Winooski, VT, USA). Using NAD⁺ and NADP⁺ standards to generate calibration curves, we interpolated sample concentrations from the standard curves.

### ROS measurement

Intracellular ROS levels were measured using the cell-permeable fluorescent probe 2',7'-dichlorodihydrofluorescein diacetate (DCFH-DA) according to the manufacturer's instructions. Intracellular esterases hydrolyze the probe to form DCFH, subsequently oxidizing it to the fluorescent product dichlorofluorescein (DCF). After washing tumor cells from different treatment groups twice with PBS, we incubated them with 5 μM DCFH-DA in serum-free medium at 37 °C for 30 minutes. After three 5-minute PBS washes, fluorescent images were acquired using an inverted epifluorescence microscope (Olympus, Japan).

### Measurement of pHi and NHE1 function

To measure intracellular pH (pHi), tumor cells were incubated with 5 μM BCECF-AM in PBS for 30 min at 37 °C. Following incubation, the cells were washed twice with fresh medium or PBS to remove unincorporated BCECF-AM. Fluorescence ratios were recorded using a Tecan M200Pro fluorescence spectrophotometer (Tecan Group Ltd., Männedorf, Switzerland) by alternately exciting the cells at 490 nm and 440 nm while detecting emissions at 530 nm. The pH values were then calculated using standard calibration curves generated from cells incubated in calibration solutions containing 10 μmol/L nigericin, as previously described 27. NHE1 activity was assessed using the NH4Cl pulse method. Briefly, acid loading was induced by treated cells with a solution containing 30 mmol/L NH4Cl (NH4^+^/NH3) for 2 min, followed by replacement with standard HEPES buffer solution, as previously described 28.

### Animal studies

PARPi and Met were administered to the animals. Mefuparib (batch no: MP20191105; stored at 4 °C) was generously provided by Pukang Pharmaceutical Co. A 50 mg/mL stock solution of mefuparib in DMSO was diluted with 0.9% NaCl to achieve a final dose of 40 mg/kg for oral gavage. Met was prepared by dissolving the powder in 0.9% NaCl solution to a final concentration of 200 mg/kg for oral administration.

Xenograft experiments were conducted using 4-6-week-old male BALB/c-nu nude mice (Beijing Vital River Laboratory Animal Technology Co., Ltd.). Prior to inoculation, PCa cells were trypsinized, washed twice with PBS, and resuspended in 100 μL PBS at a density of 4 × 10⁶ viable cells for subcutaneous injection into the dorsal region. For orthotopic implantation, a transverse incision was made in the lower abdomen of nude mice to expose the prostate, followed by an injection of 50 μL of cell suspension (4 × 10⁶ cells) into the prostatic capsule. On day 15 post-inoculation, the mice were randomly assigned to one of three treatment groups: 1. Control (saline via oral gavage) 2. Mefuparib (40 mg/kg every other day) 3. Combination therapy (mefuparib 40 mg/kg every other day + Met 200 mg/kg daily). The metformin dosing regimen (200 mg/kg daily) was selected based on its established pharmacokinetic profile in mice and its well-documented use as a classic therapeutic agent 29.

Tumor dimensions (length [L] and width [W]) were measured triweekly using digital calipers until reaching the endpoint (maximum tumor volume ~1500 mm³). Tumor volume (V) was calculated as: V = (π × L × W²)/6 Only tumors > 0.3 cm in diameter were included in statistical analyses. For orthotopic xenografts, tumor growth was monitored via bioluminescence imaging (BLI). Mice were administered an intraperitoneal injection of D-luciferin (200 mg‧kg⁻¹) dissolved in phosphate-buffered saline. After 20 minutes, the animals were anesthetized by inhalation of isoflurane, and imaging was performed using an IVIS 200 system (PerkinElmer Imaging Systems, United States). Data were processed and reconstructed using the accompanying workstation software. All procedures were approved by the Experimental Animal Ethics Committee of Tongji Hospital, Tongji Medical College, Huazhong University of Science and Technology (approval no.: T1-202406034) and complied with national regulations and the NIH Guide for the Care and Use of Laboratory Animals.

### Bulk RNA-seq analysis

Total RNA was extracted using TRIzol reagent (Sangon Biotech) according to the manufacturer's protocol. RNA sequencing libraries were prepared using the BGI RNA-Seq library preparation kit and sequenced on the DNBSEQ-T7 platform (BGI). Raw sequencing data were stored in FASTQ format.

### Statistical analysis

Categorical variables were evaluated using the Fisher's exact test, with results presented as percentages. Normally distributed continuous variables were expressed as mean ± standard deviation and analyzed using Student's t-test. Follow-up time was calculated from olaparib treatment initiation until the study cutoff date (November 2024) or patient death, whichever occurred first. Median PFS and OS were estimated using the Kaplan-Meier method. The Cox proportional hazards model was used to calculate hazard ratios (HR) and their 95% confidence intervals (CI). To identify potential confounding factors, we performed both univariate and multivariate analyses of progression-free and overall survival, incorporating baseline characteristics, treatment regimens, and genomic alterations in our models. Primary statistical analyses were performed using R (version 4.2.2) and SPSS (version 28), while secondary analyses utilized Python (version 3.10.10) with scikit-learn (1.2.2) and shiny (0.5.1) packages.

## Results

### Prostate cancer patients harboring PTEN mutations exhibit heightened sensitivity to PARP inhibitors when treated with metformin

Advanced PCa patients are predominantly elderly males 1, with diabetes prevalence reaching 38.1% in those over 70 30. This makes Met, the most common hypoglycemic agent, widely used in this population. This study retrospectively analyzed clinical data from PCa patients treated with the PARPi olaparib to investigate the combined effect of PTEN mutations and oral Met on olaparib's antitumor efficacy.

Table S1 presents baseline characteristics of all 461 olaparib-treated PCa patients. The experimental (PTEN mutation + Met, n = 50) and control (n = 411) groups showed comparable baseline parameters (Table S1), including age, PSA levels, Gleason score, pathological findings, metastatic stage, surgical history (radical prostatectomy), medication history (abiraterone treatment), and BRCA mutation status. Patients were stratified by treatment period: 279 treated during 2017-2022 underwent comprehensive evaluation (PSA50, PFS, OS), while 182 treated during 2022-2024 were assessed for PSA50 response only, pending longer follow-up for survival outcomes.

**Figure 1:**
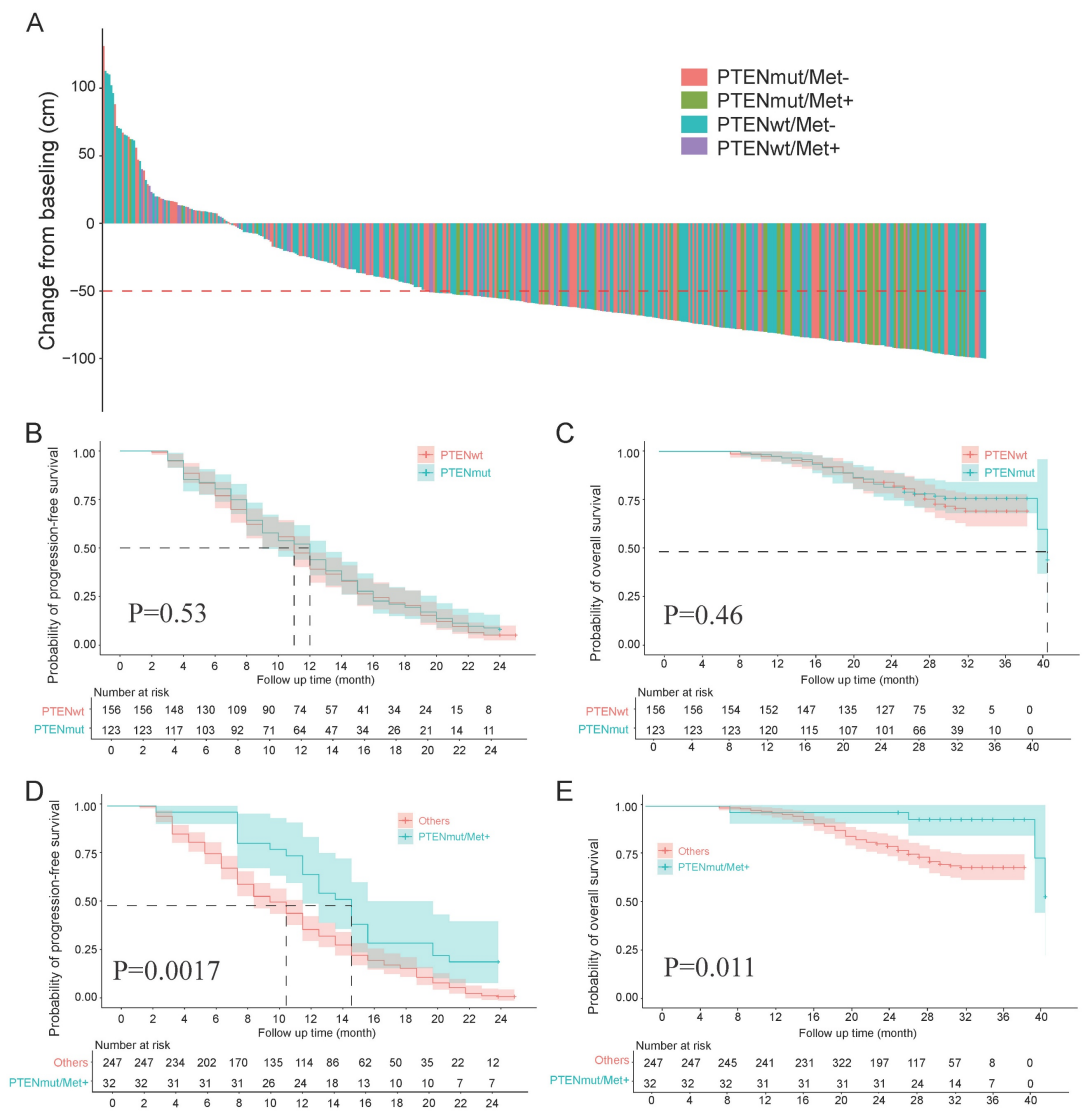
** A**, Percentage change in PSA from baseline for each patient post-treatment. **B**, **C**, Survival analysis grouped by PTEN status, comparing Kaplan-Meier curves for PFS and OS between the two groups. **D**, **E**, Survival analysis comparing Kaplan-Meier curves for PFS and OS between the experimental and control groups.

Among the 461 patients, 293 (63.6%) achieved a PSA50 response. The experimental group had a significantly higher PSA50 response rate (97.8%, 44/45) than the control group (60.6%, 249/411; p < 0.001; Table S1). PSA changes after PARPi treatment are shown in a waterfall plot (Figure 1A). Among the 411 control patients, PTEN wild-type patients had similar PSA50 response rates regardless of Met treatment (no Met: 61.2%, 139/227; with Met: 57.1%, 24/42; p = 0.618). In contrast, PTEN-mutant patients without Met treatment had a lower PSA50 response rate (58.5%, 86/43) than the experimental group (97.8%, 44/45; p < 0.001).

Analysis of PFS and OS data from 279 patients showed that 261 cases (93.5%) experienced PFS events, and 72 cases (25.8%) were associated with death. The mean PFS was 9.9 months (SD = 5.4) and mean OS was 24.5 months (SD = 5.2). In the experimental group (n = 32), The experimental group had PFS events in 25 cases (78.1%) with 4 death (12.5%), while the control group showed PFS events in 236 cases (95.5%) with 68 deaths (27.5%). While mortality event rates showed no significant difference between groups (p = 0.068), the experimental group demonstrated significantly lower PFS (78.1% vs 95.5%; p < 0.001). The experimental group showed significantly longer PFS and OSversus the control group (12.9±5.6 months vs 9.5±4.5 months; p < 0.001; 26.9±6 months vs 24.2±5 months; p = 0.006). Complete comparative data are summarized in Table S1.

In our subgroup analysis (n = 279): PTEN mutation status showed no significant association with PFS (p = 0.53) or OS (p = 0.46) (Figures 1B, C). The Met-treated and untreated groups showed comparable PFS (p = 0.23) and OS (p = 0.031) (Supplementary Figures 1A, B). PTEN mutation combined with Met treatment significantly improved PFS (p = 0.0017) and demonstrated a nonsignificant trend for OS improvement (p = 0.011) (Figures 1D, E). Among the 58 patients receiving Met treatment, the PTEN-mutated group had significantly prolonged PFS (p = 0.00082) and a trend toward prolonged OS (p = 0.011) (Supplementary Figures 1C, D). Comparisons of PFS and OS among the four patient groups stratified by PTEN status and Met treatment are shown in Supplementary Figures 1E, F.

We performed multivariate analysis using the Cox proportional hazards model to evaluate potential prognostic factors affecting PFS and OS. The results demonstrated that the combination of PTEN mutation and oral Met treatment independently predicted favorable prognosis for both PFS and OS, as detailed in Table S2. Additionally, logistic regression analyses (both univariate and multivariate) were performed to identify factors associated with PSA50 response. While univariate analysis revealed Met treatment and BRCA mutation's significant association with PSA50 response, multivariate analysis additionally identified BRCA and PTEN mutation, oral Met treatment, and distant metastasis as independent predictors. Detailed results are summarized in Table S3.

### PTEN-deficient prostate cancer exhibits PARPi resistance under lactate-enriched conditions

**Figure 2:**
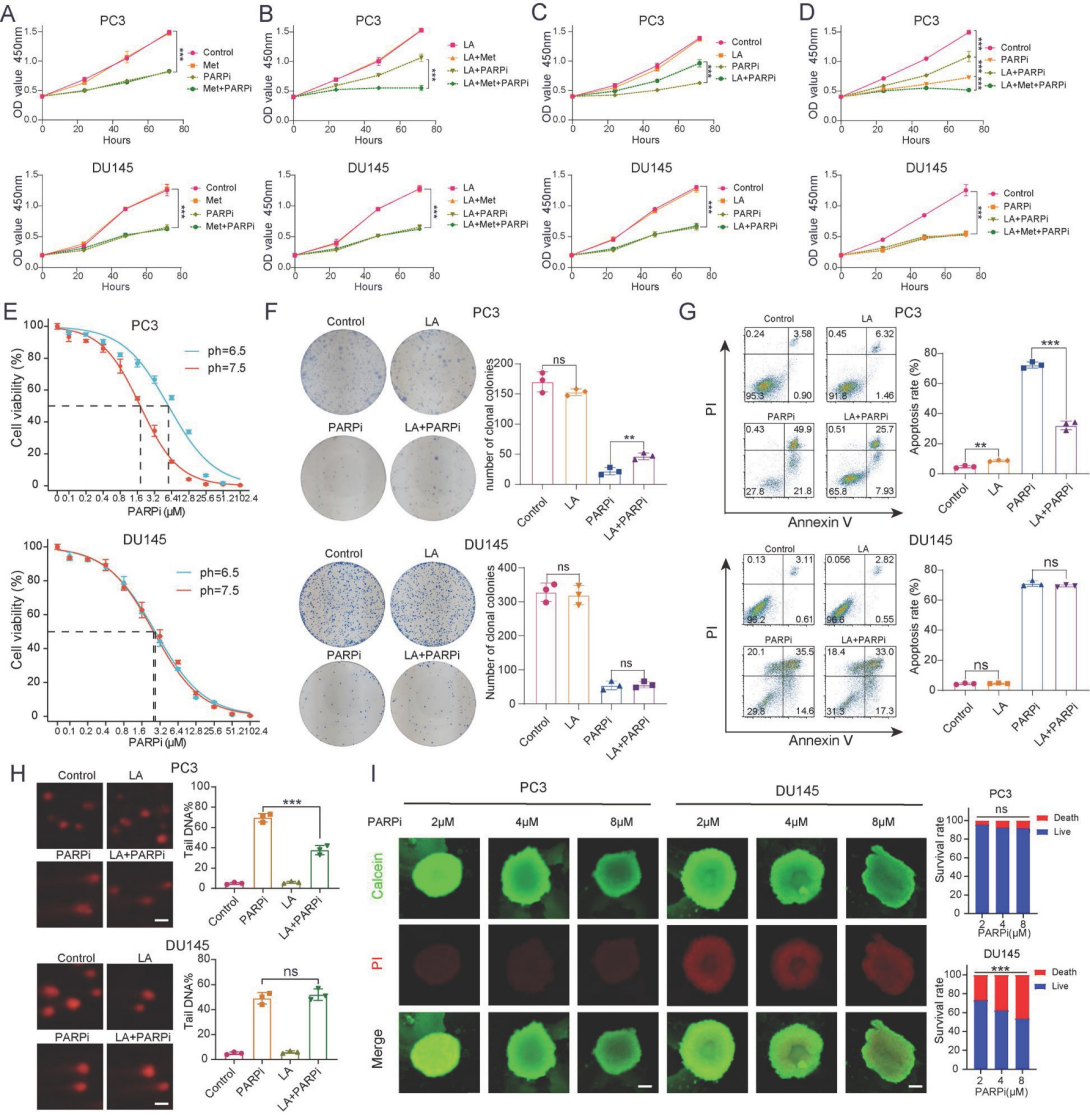
** A-D**, The proliferation of PC3 and DU145 cells was assessed using CCK-8 assay, with the following experimental groups: Control, LA, Met, and their combinations (LA+Met), as well as PARPi alone or in combination (LA+PARPi, Met+PARPi, LA+Met+PARPi). **E**, Under different pH conditions (7.5 vs. 6.5), PC3 and DU145 cells were treated with gradually increasing concentrations of PARPi for 48 hours, and cell viability was measured using the CCK-8 method to calculate IC_50_ values. **F**, Colony formation assays were performed to compare the effects of different treatments (Control, LA, PARPi, and LA+PARPi) on PC3 and DU145 cells, with quantitative results shown in the right panel. **G**, Apoptosis in PC3 and DU145 cells was assessed by flow cytometry after 48-hour treatment with Control, LA, PARPi, or LA+PARPi, and quantitative analysis of apoptotic cells is displayed in the right panel. **H**, Comet assays were used to evaluate DNA damage in PC3 and DU145 cells treated with the Control, LA, PARPi, and LA+PARPi groups (scale bar: 1 μm). **I**, PC3 and DU145 cells were seeded in ultra-low attachment 96-well plates and cultured for 48 hours before treatment with varying concentrations of mifepristone for 72 hours. Dual staining was carried out with calcein-AM (live cells, green) and propidium iodide (dead cells, red), and fluorescence images were used to assess tumor spheroid viability. Scale bar: 500 μm.

Clinical data suggest that Met (Met) enhances the antitumor effects of PARPi in PTEN-deficient prostate cancer. Previous studies indicate that Met modulates cellular functions by regulating oxidative phosphorylation (OXPHOS) metabolism 31, while tumor cells undergo metabolic reprogramming that enables switching between glycolysis and OXPHOS - a process that may promote DNA repair. To validate their synergy, we cultured cells in media containing PARPi (mefuparib) and Met. Surprisingly, CCK-8 assays revealed that Met did not significantly augment PARPi-mediated tumor cell killing (Fig. 2A). Given that lactate accumulation - a hallmark of glycolysis - has been shown to reduce olaparib sensitivity 19, since lactate accumulation is a hallmark of glycolysis, we further examined Met's impact on PARPi efficacy under lactate-enriched conditions. Results demonstrated that PTEN-deficient PC3 cells exhibited markedly stronger growth inhibition in the LA+Met+PARPi group compared to the LA+PARPi group (Fig. 2B). Notably, in the LA+PARPi group, PC3 cell growth inhibition was weaker than in the PARPi-only control (Fig. 2C). The data revealed progressively decreasing growth suppression across treatment conditions, with the LA+Met+PARPi combination showing strongest effects, followed by PARPi monotherapy, then LA+PARPi (Fig. 2D). No significant differences were observed in PTEN-wildtype DU145 cells across treatment groups (Figs. 2A-D).

These findings demonstrate that PTEN-deficient PC3 cells acquire PARPi resistance in lactate-rich environments, and this resistance is reversed by Met co-treatment. We further validated this phenomenon in PTEN-deficient LNCaP and PTEN-wildtype 22Rv1 cell lines. LNCaP cells exhibited reduced growth inhibition under LA+PARPi treatment, whereas 22Rv1 cells exhibited no growth inhibition (Supplementary Fig. 2A). To investigate whether lactate ions or acidic pH drives PARPi resistance, we prepared three media conditions simulating: (1) lactate microenvironment (LA), (2) acidic conditions (HA), and (3) lactate ion environment (SL). The pH was adjusted to 6.5 in both LA and HA groups, while SL maintained lactate ion concentration matching LA. Using CCK8 assays, we found that HA group significantly diminished PARPi efficacy against PC3 cells (Supplementary Fig. 2A), identifying acidosis as the primary driver of resistance.

Given that PARPi is primarily used clinically for CRPC 3, we selected androgen-independent PC3 and DU145 cell lines as relevant models 32. For PC3 cells, the LA group showed three-fold higher IC50 values than the Control group, whereas DU145 cells exhibited no significant differences between groups (Figure 2E). Colony formation assays revealed stronger mefuparib inhibition in PC3 cells from Control and SL groups versus LA and HA groups, while showing no differential effects across DU145 cell groups (Figure 2F, Supplementary Figure 2B). Mefuparib treatment resulted in 50% fewer apoptotic PC3 cells in LA/HA groups versus Control/SL groups (p<0.01), with DU145 cells showing no apoptosis differences across treatments (Figure 2G, Supplementary Figure 2C).

Since PARPi inhibits DNA repair to exert antitumor effects, we used the comet assay to quantify the resulting DNA damage. PC3 cells results revealed significantly less fragmented DNA in LA versus control groups, whereas DU145 cells showed no significant difference between LA and control groups (Figure 2H). Given that 3D tumor spheroids more accurately recapitulate the hypoxic gradients and cell-cell interactions of *in vivo* tumors compared to 2D cultures 33, we developed 3D models using PC3 and DU145 cells. Our quantitative analysis shows that DU145 spheroids exhibit significantly higher growth inhibition rates than PC3 spheroids at equivalent mefuparib concentrations. Dose-response analysis further showed that increasing mefuparib concentrations caused significant survival reduction in DU145 spheroids but only marginal effects in PC3 spheroids (Figure 2I).

Three PARP inhibitors (olaparib, rucaparib, and talazoparib) showed weaker growth inhibition in PC-3 and LNCaP cells from the LA group versus controls. However, DU145 and 22Rv1 cell lines showed no such difference (Supplementary Figure 2D). The experimental data suggest an acidic environment contributes to PARPi resistance in PC-3 cells, as both LA and HA (but not SL) attenuated PARPi efficacy in PC-3 and LNCaP cells. Since DU145 and 22Rv1 cells showed no resistance in acidic conditions, impaired PARPi uptake was excluded as a mechanism.

### Metformin reverses PARPi resistance in PTEN-deficient prostate cancer cells under lactate microenvironment

**Figure 3:**
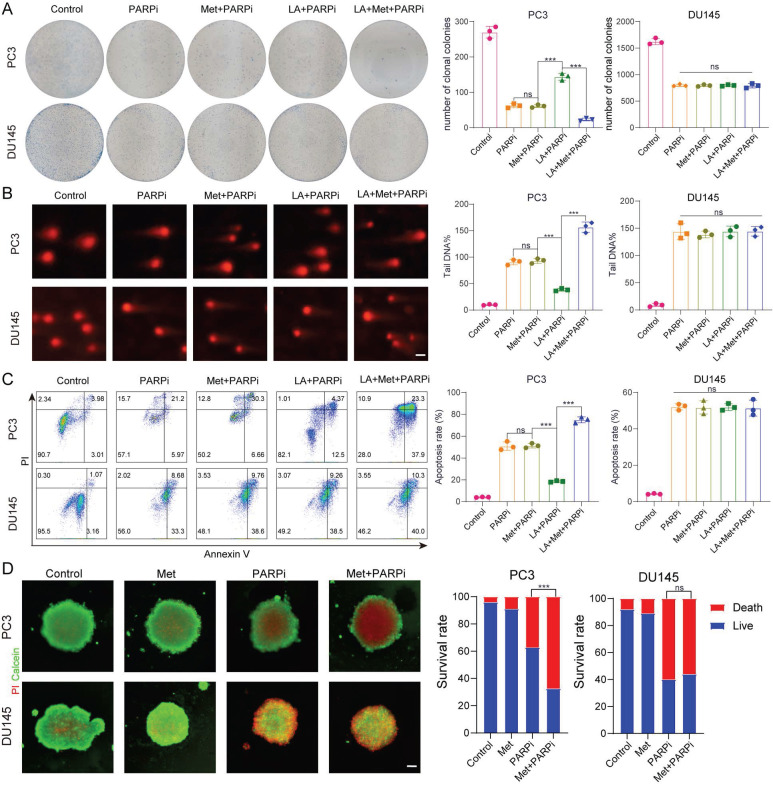
** A**, Colony formation assays were performed to assess the proliferative capacity of PC3 and DU145 cells. Colony formation was compared across five treatment groups: Control, PARPi, Met+PARPi, LA+PARPi, and LA+Met+PARPi. **B**, DNA damage in PC3 and DU145 cells was evaluated by comet assay following treatment with Control, PARPi, Met+PARPi, LA+PARPi, or LA+Met+PARPi. Quantitative analysis of DNA damage is shown in the right panel. Scale bar: 1 μm. **C**, After 48-hour treatment with Control, PARPi, Met+PARPi, LA+PARPi, or LA+Met+PARPi, apoptosis in PC3 and DU145 cells was assessed by flow cytometry. The quantification of apoptotic cells was presented in the right panel. **D**, PC3 and DU145 cells were seeded in ultra-low-attachment 96-well plates for 48 hours, then treated for 72 hours with Control, PARPi, Met, or Met+PARPi. After dual staining with calcein-AM (green fluorescence indicating live cells) and propidium iodide (PI; red fluorescence indicating dead cells), fluorescence images were acquired. Tumor spheroid fluorescence ratios were quantified using ImageJ (right panel). Scale bar: 500 μm.

Based on the above findings, we further investigated the effect of Met on PARPi efficacy in PC-3 and DU-145 cells under different microenvironments. Through colony formation, comet, and apoptosis assays, we consistently observe that Met does not affect the antitumor effects of PARPi in PC-3 and DU-145 cells under normal conditions. Interestingly, in lactate-enriched conditions, while Met abolished PARPi resistance and enhanced antitumor effects in PC-3 cells, DU-145 cells showed neither response (Fig. 3A-C). 3D tumor spheroid assays confirmed that Met potentiated the cytotoxic effects of PARPi in PC-3 cells but had no impact on DU-145 cells (Fig. 3D).

Based on the aforementioned findings, we sought to determine whether PTEN regulates intracellular lactate production through modulation of metabolic pathways, thereby influencing the sensitivity of prostate cancer cells to PARP inhibitors. To investigate this, we generated stable PTEN-knockdown DU145 cells (DU145-PTEN-KD) using lentiviral delivery of shRNA. RNA sequencing (RNA-seq) analysis identified substantial transcriptional reprogramming in DU145-PTEN-KD cells compared to control cells, with 3,173 genes significantly upregulated and 2,248 genes downregulated (|log₂ fold change| > 2, FDR < 0.05; Supplementary Fig. 3A).Gene set enrichment analysis (GSEA) revealed significant enrichment of lactate metabolism-related pathways in DU145-PTEN-KD cells (Supplementary Fig. 3B,C), indicating that PTEN loss enhances lactate biosynthesis and efflux. Consistent with this finding, Gene Ontology (GO) analysis of differentially expressed genes showed marked upregulation of terms including "NAD⁺ nucleosidase activity" and "NAD(P)⁺ nucleosidase activity," suggesting an increase in NAD⁺ catabolism that may drive redox imbalance and support metabolic adaptation in PTEN-deficient prostate cancer cells (Supplementary Fig. 3D).

### Intracellular NADP^+^ affects the tumor toxicity of PARP inhibitors

**Figure 4:**
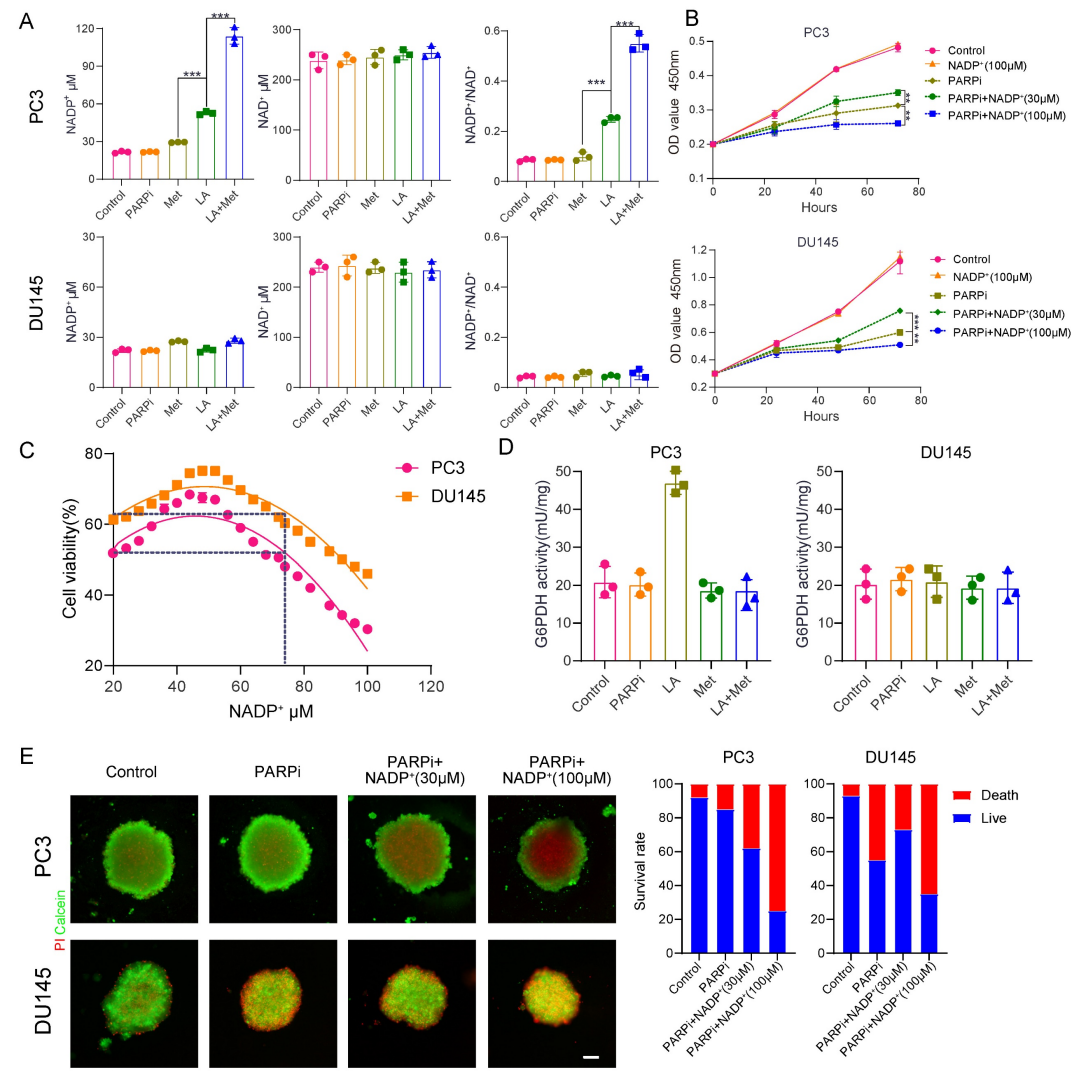
**A**, Measurement of NAD⁺ and NADP⁺ concentrations and the NADP⁺/NAD⁺ ratio in PC3 and DU145 cells under Control, PARPi, Met, LA, and LA+Met conditions. **B**, Proliferation of PC3 and DU145 cells in Control, NADP⁺ (100 mM), PARPi, PARPi + NADP⁺ (30 mM), and PARPi + NADP⁺ (100 mM) groups, assessed by CCK-8 assay. **C**, CCK-8 assay to quantify the IC₅₀ of PARPi in PC3 and DU145 cells under different NADP⁺ concentrations. **D**, Colorimetric assay to measure glucose-6-phosphate dehydrogenase (G6PDH) enzyme activity in cells (grouped as in A). **E**, PC3 and DU145 cells cultured in ultra-smooth 96-well plates for 48 h, followed by 72 h treatment in Control, PARPi, PARPi + NADP⁺ (30 mM), and PARPi + NADP⁺ (100 mM) groups. Fluorescence images were captured after calcein-AM (green, live cells) and propidium iodide (PI, red, dead cells) staining. Tumor spheroid fluorescence ratios were quantified using ImageJ (right panel). Scale bar: 500 μm.

We still don't understand how the lactic acid microenvironment and Met regulate PARPi sensitivity in PC3 cells. Given the structural similarity between PARPi and NAD^+^, and the role of intracellular NADP^+^ as an endogenous PARPi that significantly influences its antitumor effects 23, this study examines whether LA and Met modulate PARPi's antitumor efficacy through NADP^+^ level regulation. Quantification of NADP^+^ and NAD^+^ concentrations in PC3 and DU145 cells across Control, PARPi, Met, LA, and LA+Met groups showed significantly increased NADP^+^ levels only in PC3 cells treated with LA or LA+Met. The LA group showed an NADP^+^ concentration of 50.2 ± 3.1 μM, while the LA+Met group reached 119.8 ± 5.4 μM. Notably, this effect was absent in DU145 cells, and NAD^+^ levels remained stable across all treatments in both cell lines. Correspondingly, the NADP^+^/NAD^+^ ratio in PC3 cells was 0.1 in Control, PARPi, and Met groups, increasing to 0.25 in the LA group and further to 0.5 in LA+Met. In contrast, DU145 cells maintained a ratio of 0.1 across all treatments (Fig. 4A).

To investigate the impact of NADP^+^ concentration changes on PARPi sensitivity, we conducted CCK8 assays. The CCK8 assays revealed that PC3 and DU145 cells developed resistance to mefuparib at 30 μM extracellular NADP^+^, while sensitivity significantly increased at 100 μM (Fig. 4B). Furthermore, mefuparib's IC50 values in both cell lines followed a biphasic pattern as intracellular NADP^+^ levels increased, peaking at approximately 50 μM. Resistance to mefuparib peaked at this concentration (50 μM) before gradually declining, with complete resistance loss occurring above 80 μM as cells transitioned to a sensitive state (Fig. 4C).

In 3D tumor spheroid assays, as extracellular NADP^+^ increased from 30 μM to 100 μM, PARPi's cytotoxic effect on PC3 cells progressively intensified, whereas DU145 cells initially exhibited resistance before becoming more sensitive (Fig. 4E). Further analysis showed significantly different NADP^+^ levels between PC3 and DU145 spheroids (Supplementary Fig. 4A): baseline NADP^+^ was ~50 μM in PC3 compared to ~20 μM in DU145 controls. At 30 μM and 100 μM extracellular NADP^+^, PC3 cells showed progressive PARPi sensitivity enhancement, while DU145 cells transitioned from resistance to sensitivity.

LA and Met regulate intracellular NADP^+^ through generation and elimination processes. The NADP^+^ oxidase (NOX) protein family primarily regulates NADP^+^ production while simultaneously generating reactive oxygen species (ROS) 34. This study found significantly higher ROS secretion in PC3 cells treated with LA versus Control, but no difference between LA and LA+Met treatments. DU145 cells exhibited similar ROS levels among Control, LA, and LA+Met treatments (Supplementary Fig. 4B). Our findings suggest LA enhances NADP^+^ synthesis in PC3 cells by activating NOX proteins. In contrast, Met appears to function independently of NOX regulation, potentially by preventing NADP^+^ depletion.

Cells reduce NADP^+^ via the pentose phosphate pathway (PPP), where glucose-6-phosphate dehydrogenase (G6PDH) catalyzes its conversion to NADPH 35, Prior studies indicate Met inhibits G6PDH 36, and our work further examined its activity in PC3 and DU145 cells under different conditions. In PC3 cells, G6PDH showed a significant increase in the LA group versus control, likely due to NADP^+^ driven feedback activation. The G6PDH activity in the LA+Met group was lower than that in the LA group, whereas no difference existed between control and Met-alone groups. Conversely, DU145 cells showed no G6PDH activity changes across treatments (Fig. 4D). These findings demonstrate that Met preferentially inhibits hyperactivated G6PDH over basal activity, explaining why NADP^+^ levels in Met-treated PC3 and DU145 cells matched those in controls.

Since ROS influences tumor growth to some extent 37, to clarify whether ROS modulates the antitumor effects of PARPi, we validated this through both exogenous ROS supplementation and intracellular ROS scavenging. Galactose oxidase (GO) and D-galactose (GA) can generate H₂O₂ in the culture medium, which rapidly enters the cytoplasm 38. Concurrently, NAC treatment effectively reduced intracellular ROS levels 39 (Supplementary Fig. 4B, C). The CCK-8 assay further verified the impact of ROS on PARPi, showing that neither increasing nor decreasing ROS levels in PC3 and DU145 cells altered their sensitivity to mevuparib (Supplementary Fig. 4D). Consistent with the CCK-8 results, apoptotic and comet assays revealed that ROS level alterations did not influence PARPi resistance in PC3 and DU145 cells. Notably, 30 μM NADP⁺ induced mevuparib resistance, while 100 μM NADP⁺ paradoxically enhanced drug sensitivity (Supplementary Fig. 4E, F).

### PTEN inhibits NADP^+^ oxidase 1 (NOX1)

**Figure 5:**
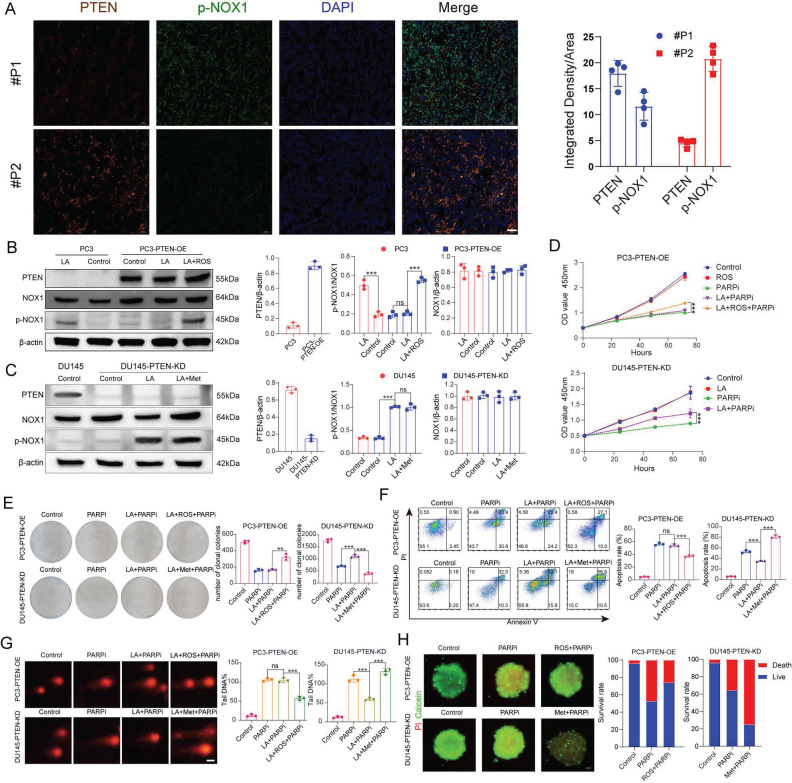
** A**, Immunofluorescence analysis of prostate cancer tissue sections shows PTEN (red), p-NOX (green), and DAPI (blue) staining in tumor cells (left). Scale bar: 50 μm. Quantitative analysis is shown (right). **B**, **C**, Western blot (WB) was used to detect PTEN, NOX, p-NOX, and β-actin protein levels in PC3 and PC3-PTEN-OE cells under Control, LA, and LA+ROS conditions, as well as in DU145 and DU145-PTEN-KD cells under Control, LA, and LA+Met conditions. Band density quantification is shown (right). **D**, CCK-8 assay was used to measure the proliferation rate of PC3-PTEN-OE and DU145-PTEN-KD cells under different treatment conditions. **E**, Colony formation assay shows the clonogenic ability of PC3-PTEN-OE and DU145-PTEN-KD cells under different treatments, with colony quantification shown (right). **F**, PC3-PTEN-OE and DU145-PTEN-KD cells were treated under different conditions for 48 hours, and apoptosis was detected by flow cytometry. The right panel shows quantitative apoptosis analysis. **G**, Comet assay was used to assess DNA damage in PC3-PTEN-OE and DU145-PTEN-KD cells under different treatments, with DNA damage quantification shown (right). Scale bar: 1 μm. **H**, PC3-PTEN-OE and DU145-PTEN-KD cells were cultured in ultra-low-attachment 96-well plates for 48 hours, followed by treatment in Control, PARPi, ROS+PARPi, and Met+PARPi media for 72 hours. Fluorescence images were captured after calcein-AM (green, live cells) and PI (red, dead cells) staining. Tumor spheroid fluorescence ratios were measured using ImageJ (right). Scale bar: 250 μm.

Under lactate treatment, NADP^+^ and ROS levels increased in PC3 cells but remained unchanged in DU145 cells. We therefore hypothesized that NOX proteins are activated in PC3 cells under lactate conditions. In DU145 cells, however, certain factors appear to inhibit NOX activation. To test this hypothesis, we obtained pathological specimens from prostate cancer patients at Tongji Hospital, and performed immunofluorescence staining. The results revealed an inverse correlation between PTEN and p-NOX protein expression (Figure 5A). This finding supports the idea that PI3K inhibitors can enhance the anti-tumor effect of PARPi in PTEN deficient prostate cancer 40.

This study aimed to further validate PTEN's critical role in regulating PARPi sensitivity. We constructed PTEN-overexpressing PC3 cells (PC3-PTEN-OE) and PTEN-knockdown DU145 cells (DU145-PTEN-KD). Western blot analysis showed increased phosphorylated NOX (p-NOX) expression in PC3 cells under lactate conditions, while PC3-PTEN-OE cells showed no significant change. Given that ROS oxidizes and inhibits PTEN function 41, we observed elevated p-NOX expression in PC3-PTEN-OE cells when ROS was added to lactate conditions (Figure 5B). In DU145 cells, p-NOX expression remained unaltered in the lactate microenvironment; however, in DU145-PTEN-KD cells, The LA medium markedly increased p-NOX expression, with no significant difference between LA+Met and LA medium (Figure 5C, Supplementary Figure 5B).

PC3-PTEN-OE and DU145-PTEN-KD cells showed opposite resistance patterns to mefuparib compared to their wild-type counterparts. Using multiple assays (CCK8 proliferation, colony formation, comet, apoptosis, and 3D tumor spheroid), we found that PC3-PTEN-OE cells in the LA group showed no resistance to PARPi, while combined LA+ROS treatment reinstated resistance. Conversely, DU145-PTEN-KD cells maintained PARPi resistance with LA treatment but showed restored sensitivity in the LA+Met group (Figure 5D-H). Notably, NADP^+^ levels remained unchanged in LA-treated PC3-PTEN-OE cells, whereas LA+ROS treatment induced a significant increase. In DU145-PTEN-KD cells, compared with the control group, the NADP^+^ production in the LA group increased by 125%, while the LA+Met group increased by 350% (Supplementary Figure 5F).

This study examines if ROS-mediated PTEN suppression regulates NOX protein-dependent NADP^+^ generation. While NADP^+^ levels remained unchanged in LA-only or ROS-only treated DU145 cells, the LA+ROS combination treatment significantly increased NADP^+^ levels (Supplementary Figure 5A). WB results showed upregulated p-NOX expression in LA+ROS-treated DU145 cells, while neither LA nor ROS treatment alone elevated p-NOX levels (Supplementary Figure 5B). Both comet assays and apoptosis assays showed that DU145 cells developed PARPi resistance when cultured in LA+ROS medium (Supplementary Figures 4C, D). In the ROS group, DU145 tumor spheroids also showed PARPi resistance (Supplementary Figure 5E). Collectively, while ROS alone induces PTEN inhibition, its synergistic interaction with lactate is required for NOX1 activation and NADP^+^ generation.

### Lactic acid secretes NADP^+^ by activating NHE1/NOX pathway

**Figure 6:**
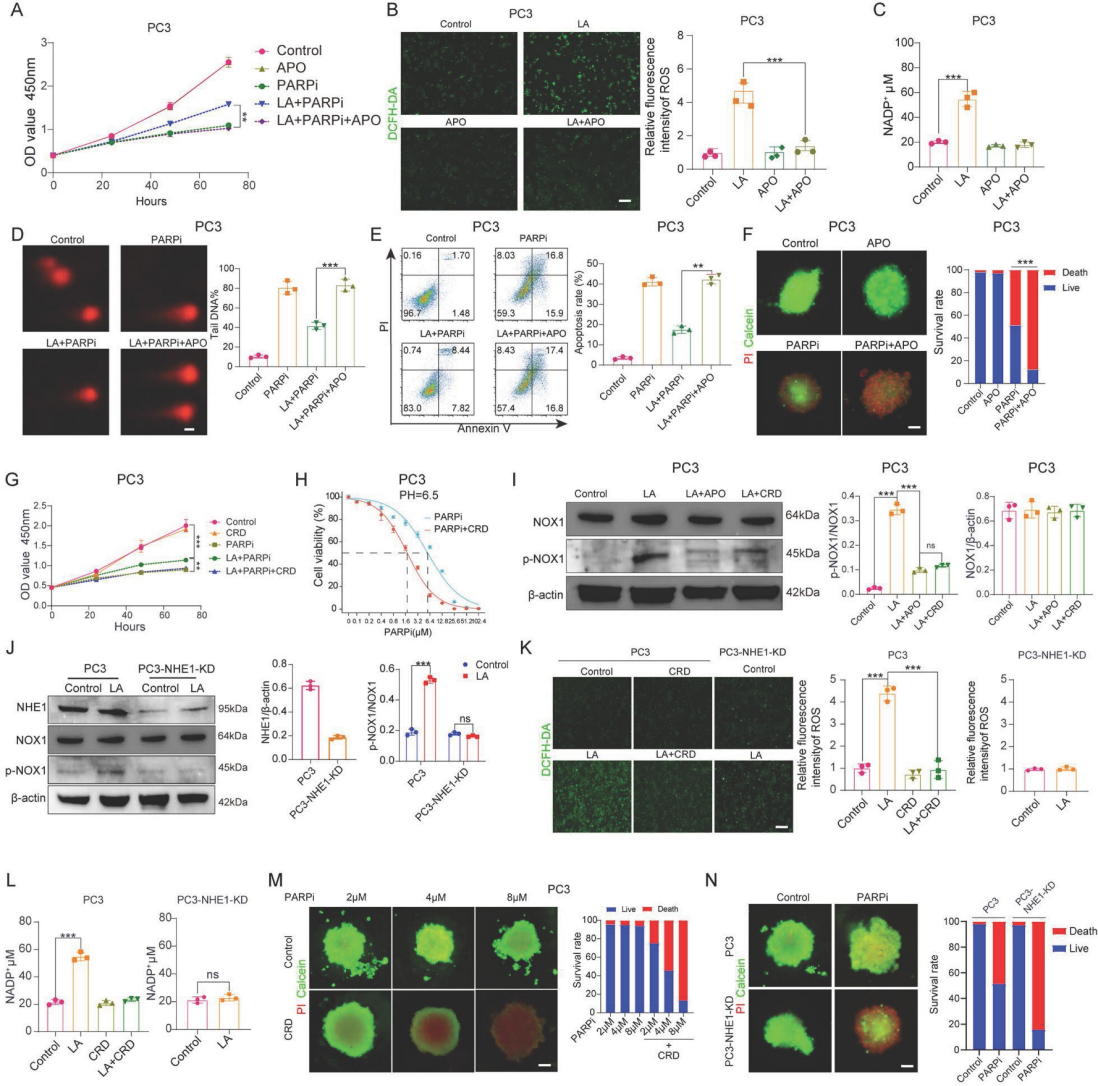
**A**, PC3 cell proliferation was evaluated using the CCK-8 assay, comparing differences among the Control, APO, PARPi, LA+PARPi, and LA+PARPi+APO groups. **B**, ROS generation in PC3 cells from the Control, LA, APO, and LA+APO groups was detected by laser confocal microscopy (left), followed by quantitative analysis (right). **C**, NADP^+^ secretion levels in PC3 cells from the Control, LA, APO, and LA+APO groups (right). **D**, DNA damage in PC3 cells from the Control, PARPi, LA+PARPi, and LA+PARPi+APO groups was measured via the comet assay, with quantitative results shown (right). Scale bar: 1 μm. **E**, Apoptosis in PC3 cells treated for 48 hours in the Control, PARPi, LA+PARPi, and LA+PARPi+APO groups was analyzed by flow cytometry, with quantitative results displayed (right). **F**, After 48 hours of culture in ultra-low attachment 96-well plates, PC3 cells were treated for 72 hours with Control, APO, PARPi, or PARPi+APO, followed by Calcein-AM/PI dual-staining fluorescence imaging. Tumor spheroid fluorescence ratios were quantified using ImageJ (right). Scale bar: 500 μm. Calcein-AM (green: live cells); PI (red: dead cells). **G**, PC3 proliferation in the Control, CRD, PARPi, LA+PARPi, and LA+PARPi+CRD groups was quantified using the CCK-8 assay. **H**, The IC50 of PARPi in PC3 cells treated with LA or LA+CRD was determined by the CCK-8 assay. **I**, Western blot (WB) analysis of NOX, p-NOX, and β-actin protein levels in PC3 cells from the Control, LA, LA+APO, and LA+CRD groups, with band density quantification (right). **J**, WB analysis of NHE1, NOX1, p-NOX, and β-actin protein levels in PC3 and PC3-NHE1-KD cells from the Control and LA groups, with band density quantification (right). **K**, ROS secretion in PC3 cells from the Control, LA, CRD, and LA+CRD groups, as well as in PC3-NHE1-KD cells from the Control and LA groups, was assessed by laser confocal microscopy (left), with quantitative ROS analysis (right). **L**, NADP^+^ secretion levels in PC3 cells from the Control, LA, CRD, and LA+CRD groups, as well as in PC3-NHE1-KD cells from the Control and LA groups (right). **M**, **N**, PC3 and PC3-NHE1-KD cells were cultured in ultra-low attachment 96-well plates for 48 hours, followed by 72-hour treatment with varying concentrations of mefuparib and CRD. Calcein-AM/PI dual-staining fluorescence imaging was performed, and tumor spheroid fluorescence ratios were quantified using ImageJ (right). Scale bar: 500 μm. Calcein-AM (green: live cells); PI (red: dead cells).

It has been confirmed that NADP^+^ production in PC3 cells under lactate-enriched conditions is primarily driven by NOX1 protein activation. CCK-8 assays validated NOX1's significance. Adding the NOX inhibitor apocynin (APO) to lactate-containing medium significantly reduced PC3 cell resistance to mefuparib (Figure 6A). The LA+APO group showed markedly decreased ROS and NADP^+^ levels in PC3 cells, while the APO-only group showed no significant changes (Figures 6B, C). Three independent assays (comet, apoptosis, and single-cell colony formation) confirmed LA+APO group PC3 cells lost PARPi resistance (Figures 6D, E; Supplementary Figure 6A). Additionally, the PARPi+APO group showed significantly higher PC3 tumor spheroid mortality than the PARPi-only group (Figure 6F).

We investigated how lactate activates NOX1 by examining the role of Na^+^/H^+^ exchanger 1 (NHE1). NHE1, a membrane transport protein activated in acidic microenvironments, shows upregulated expression and activity in malignant tumors 42. Previous studies have also linked NHE1 to tumor drug resistance 43, The NHE1/PKC/NOX signaling axis activates NOX to promote NADP^+^ production 44. CCK8 assays showed that Cariporide (CRD, 50 μM) 45, an NHE1 inhibitor, reversed PC3 cell resistance to mefuparib in lactate-containing medium (Figure 6G), but had no significant effect on DU145 cells (Supplementary Figure 6B). The NHE1 inhibitor decreased the IC50 of mefuparib against lactate-treated PC3 cells by 66% (Figure 6H), with no significant effect on DU145 cells (Supplementary Figure 6C).

In this study, we created PC3-NHE1-KD and DU145-NHE1-KD cell lines with substantially lower NHE1 protein levels than wild-type cells (Supplementary Figure 6D). CCK8 assays revealed no significant difference in mefuparib-induced growth inhibition between the Control and LA groups for PC3-NHE1-KD cells (Supplementary Figure 6G). Since NHE1 functions as a sodium-hydrogen exchanger that activates during acidosis to remove intracellular H^+^ and regulate pH, we assessed its activity through intracellular pH monitoring 46. We measured intracellular pH (pHi) recovery in: (1) wild-type PC3 and DU145 cells, (2) NHE1-knockdown lines (PC3-NHE1-KD and DU145-NHE1-KD), and (3) CRD-treated wild-type cells following NH4Cl prepulse-induced acidification. The results indicated that NHE1 functioned normally in PC3 and DU145 cells, whereas either CRD treatment or NHE1 knockdown impaired NHE1 activity, significantly attenuating pHi recovery (Supplementary Figure 6E). Using three complementary approaches (colony formation, apoptosis, and comet assays), we found that PC3 cells lost their PARPi resistance when cultured in LA+CRD medium. DU145 cells showed comparable PARPi sensitivity whether cultured in LA, LA+CRD, or standard PARPi medium. Furthermore, PC3-NHE1-KD cells showed comparable PARPi sensitivity between the LA and Control groups (Supplementary Figures 6F, H, I, J).

After confirming the critical role of the NHE1 protein, we further investigated the interaction between NHE1 and NOX proteins. Western blot analysis showed significantly lower p-NOX expression in PC3 cells treated with either LA+APO or LA+CRD, compared to LA treatment alone (Figure 6I). No significant difference in p-NOX expression was observed between the Control and LA groups in PC3-NHE1-KD cells (Figure 6J). Both PC3 cells treated with LA+CRD and PC3-NHE1-KD cells treated with LA showed intracellular ROS and NADP^+^ levels comparable to Control conditions (Figures 6K, L). Mefuparib showed enhanced inhibition of PC3 tumor spheroids when combined with CRD (Figure 6M). Furthermore, at equivalent concentrations, mefuparib inhibited PC3-NHE1-KD spheroids more effectively than wild-type PC3 spheroids (Figure 6N).

### Animal experiments demonstrated that PTEN-deficient prostate cancer is more sensitive to Met+PARPi

To investigate the efficacy of Met combined with PARPi in different prostate cancer models, we established both subcutaneous and orthotopic xenograft tumor models in nude mice. After successful tumor engraftment (approximately 14 days post-inoculation), the mice were randomized into three groups: control, PARPi monotherapy, and combination therapy. The control received 0.9% saline (oral gavage), the PARPi-only group received 50 mg/kg PARPi (every other day), while the combination group received both PARPi (same regimen) plus 200 mg/kg Met (daily).

**Figure 7:**
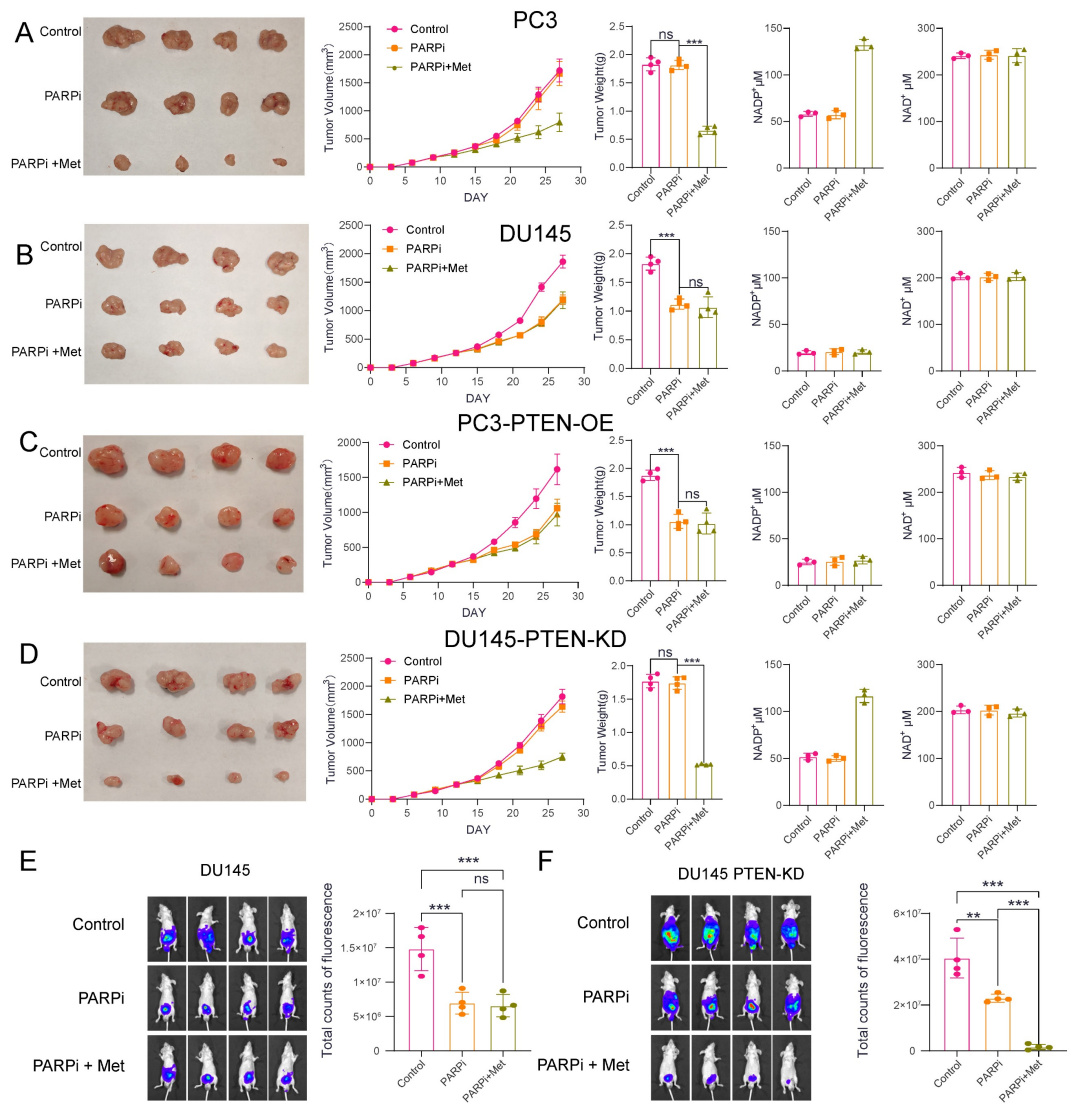
** A-D**, We established tumor xenograft models through subcutaneous inoculation of four cell lines (PC3, DU145, PC3-PTEN-OE, and DU145-PTEN-KD) into nude mice. The control group received 0.9% sodium chloride solution via intraperitoneal perfusion, while the PARPi group received PARP inhibitor (50 mg/kg, oral administration every other day). The PARPi+Met group received both PARP inhibitor (50 mg/kg, every other day) and Met (200 mg/kg, daily), both administered orally. We evaluated treatment efficacy using three methods: (1) serial digital photography for tumor size documentation, (2) tumor volume growth curves, and (3) post-sacrifice tumor weight measurements. To investigate potential metabolic mechanisms, we quantified intratumorally concentrations of NAD^+^ and NADP^+^.** E,F** Orthotopic prostate tumor models were established in nude mice via subcapsular inoculation of two cell lines: DU145 and DU145-PTEN-KD. Mice were treated with the same three therapeutic regimens as described previously. Representative *in vivo* bioluminescence images and quantitative comparison of fluorescence intensity across the three groups are shown.

Notably, the results demonstrated cell-line-specific responses, in PC3 and DU145-PTEN-KD cell lines, PARPi alone showed limited efficacy, but its antitumor effect was significantly enhanced when combined with Met. Following Met treatment, NADP^+^ levels in PC3 and DU145-PTEN-KD tumors increased from 55 μM (without Met) to approximately 130 μM (with Met) (Figures 7A, D). In DU145 and PC3-PTEN-OE cell lines, PARPi effectively suppressed tumor growth, but Met addition provided no further antitumor benefit. NADP^+^ levels in DU145 and PC3-PTEN-OE tumors maintained consistent levels (~25 μM) with or without Met treatment (Figure 7B, C).

To better mimic the tumor microenvironment of prostatecancer, we further analyzed orthotopic xenograft mouse models of prostate cancer. The results demonstrated that in DU145 cells, PARPi treatment alone inhibited tumor growth; however, the addition of metformin (Met) did not enhance the antitumor efficacy of PARPi. In contrast, in the DU145-PTEN-KD cell line, Met significantly potentiated the antitumor effect of PARPi, leading to effective suppression of tumor growth (Fig. 7E, F).

### Mechanism of NADP^+^ regulating PARPi cytotoxicity at different concentrations

**Figure 8:**
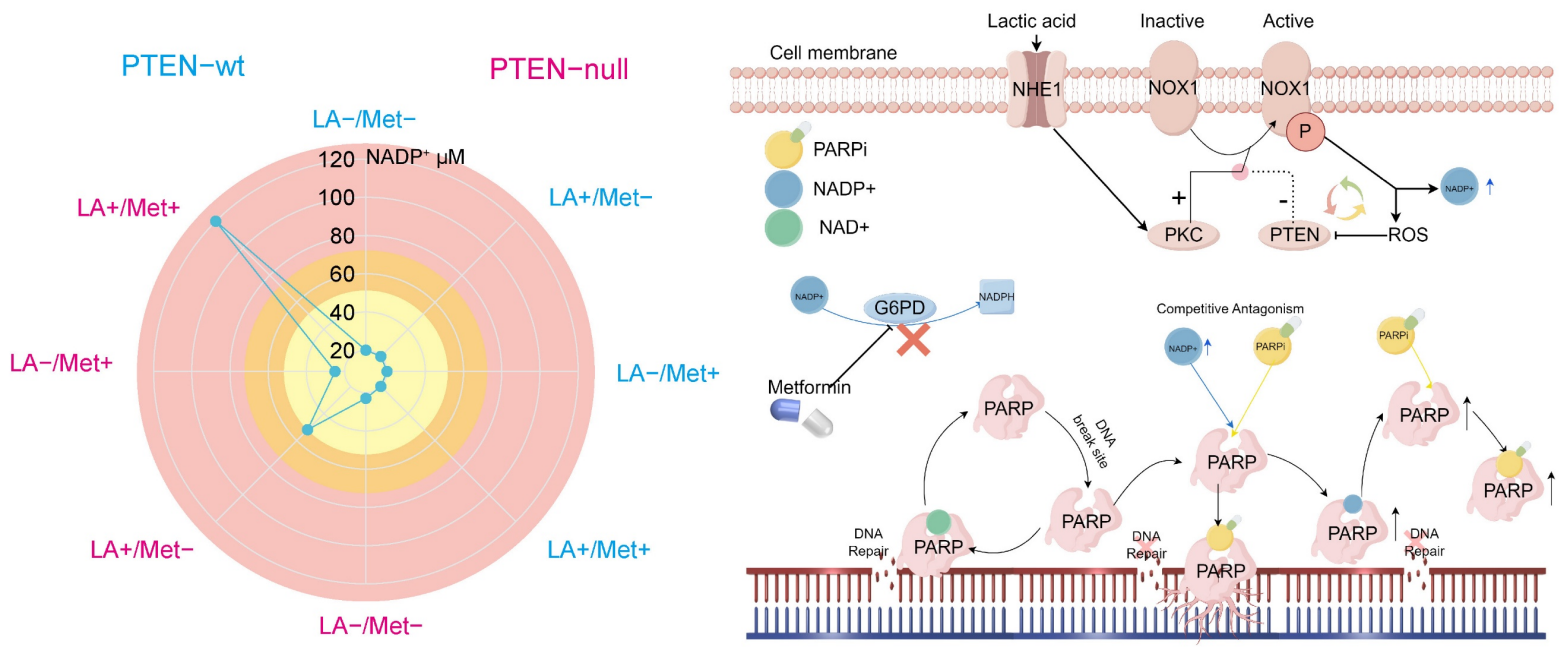
** A**, The schematic diagram illustrating the effects of NADP^+^ concentration on PARPi sensitivity demonstrates that within the low NADP^+^ concentration range (yellow), an increase in NADP^+^ concentration correlates with enhanced cellular resistance to PARPi. Conversely, in the intermediate concentration range (orange-yellow), elevated NADP^+^ concentrations lead to reduced cellular resistance to PARPi. Ultimately, in the high concentration range (pink), rising NADP^+^ concentration markedly increases cellular sensitivity to PARPi. **B**, The lactic acid microenvironment first activates NHE1 protein on the cell membrane surface, which then triggers PKC-mediated activation of NOX protein. Meanwhile, PTEN negatively regulates NOX protein. When activated, NOX1 produces NADP^+^ and ROS, with ROS negatively regulating PTEN. PTEN deficiency creates a self-reinforcing cycle: reduced PTEN allows more ROS production, which further inhibits PTEN, leading to substantial NADP^+^ and ROS accumulation. The simultaneous activation of these two pathways promotes NOX protein to produce abundant NADP^+^. Met controls NADP^+^ levels through G6PD inhibition, thereby blocking NADP^+^ reduction. Both NADP^+^ and PARPi share structural similarities with NAD^+^ and can bind to PARP1 to inhibit DNA repair. As structural analogs competing for PARP1 binding sites, NADP^+^ and PARPi exhibit concentration-dependent antagonism: moderate NADP^+^ preferentially binds PARP1, displacing PARPi, whereas excessive NADP^+^ opens numerous PARP binding sites, forming abundant PARP-PARPi complexes that suppress DNA repair.

Our data show that intracellular NADP^+^ levels regulate PARPi efficacy. Cellular resistance to PARPi follows a parabolic trend relative to NADP^+^ concentration. Resistance peaks at approximately 50 μM, then declines correspondingly, disappearing beyond 70 μM, at which point cells regain sensitivity to PARPi. After that, the sensitivity gradually increased (Figure 8).

In prostate cancer cells, NADP^+^ production is regulated by multiple pathways, with NOX1 protein playing a key role in mediating its biosynthesis. Notably, NHE1 protein positively regulates NOX1 activity, while PTEN protein exerts negative regulation. During acidosis with PTEN deficiency, NHE1 activation markedly boosts NOX1 activity, promoting substantial NADP^+^ synthesis. Cells normally maintain NADP^+^/NADPH balance through NADPH generation in the pentose phosphate pathway. However, Met inhibits the key enzyme G6PD, blocking this reduction, leading to NADP^+^ accumulation—reaching up to 120 μM in PC3 cells treated with LA+Met.

In its resting state, PARP protein maintains a closed binding site conformation that prevents NAD^+^ binding and subsequent DNA repair activation. After DNA damage occurs, PARP structurally rearranges. This change allows NAD^+^ to be used for PAR chain formation and facilitates DNA repair 47. Both PARPi and NADP^+^ competitively inhibit PARP by binding to its active site, thereby blocking NAD^+^-dependent repair mechanisms. To illustrate this competitive binding, we performed molecular docking simulations visualizing the interactions of three PARPi compounds and NADP⁺ with the PARP active site (Supplementary Figure 7A). However, PARPi has an additional mechanism called PARP-trapping: it induces conformational changes that stabilize PARP-DNA damage complexes, leading to persistent repair inhibition. No experimental evidence shows that NADP^+^ has this PARP-trapping mechanism. Therefore, moderately increasing NADP^+^ levels could competitively counteract PARPi effects by reduce PARP trapping.

To further validate the competitive inhibitory relationship between NADP⁺ and PARPi, this study investigated three PARP inhibitors with distinct PARP-binding affinities: Talazoparib, olaparib, and niraparib. Among them, Talazoparib exhibits a PARP-binding affinity approximately 100-fold greater than that of Olaparib 48. We employed CCK-8 assays, flow cytometry-based apoptosis analysis, colony formation assays, and comet assays to evaluate the influence of NADP⁺ on the activity of these PARPis (Supplementary Figure 7B-D). Our results indicate that at lower concentrations, NADP⁺ attenuates the efficacy of all three PARPis, whereas at higher levels, it appears to enhance their antitumor effects. Furthermore, due to the differing binding affinities of these inhibitors toward PARP, we observed that as the binding affinity of the PARPi increases, the modulatory effect of NADP⁺ gradually weakens. These findings are consistent with our proposed model in which NADP⁺ modulates PARP activity through competitive binding and induced conformational changes. The mechanism by which elevated NADP^+^ levels increase PARPi sensitivity remains unclear. While prior studies have confirmed this phenomenon 23, they offered no mechanistic explanation. Since NAD^+^ analogs can induce conformational changes in PARP's catalytic domain 47, we hypothesize that NADP^+^ binding may induce PARP conformational changes that maintain an open binding site. In this state, elevated NADP^+^ concentrations could both activate multiple PARP molecules. These activated PARPs form stable complexes with inhibitors, depleting functional PARPs and compromising DNA repair (Figure 8).

### Establishment of interpretive analysis and predictive models

**Figure 9:**
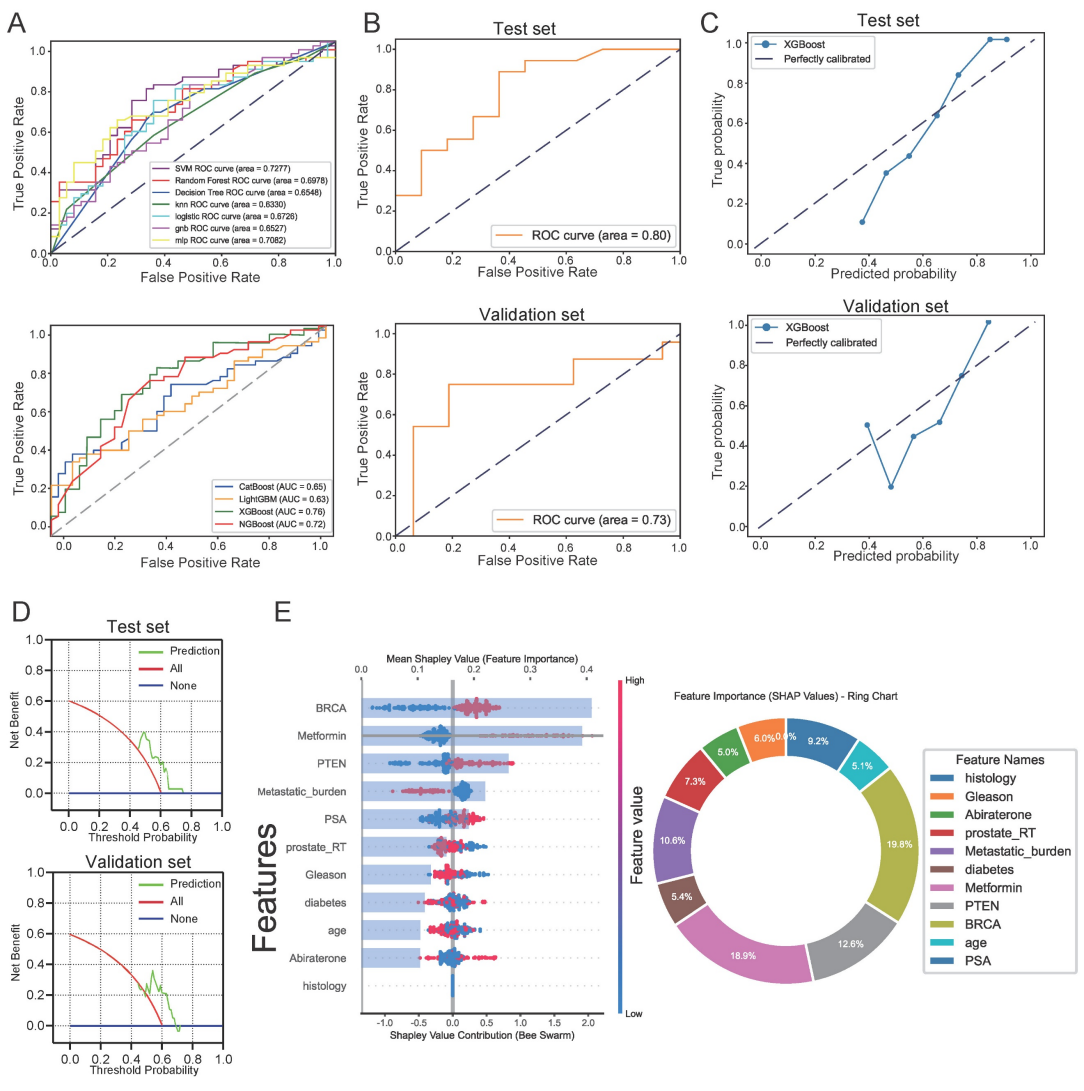
Evaluation of Machine Learning Model Predictions. **A**, ROC curve comparisons: Top panel shows seven baseline models (Decision Tree SVM, KNN, Random Forest, Logistic Regression, GNB, MLP, and Random Forest); Bottom panel displays four boosting ensemble methods (LightGBM, XGBoost, CatBoost, and NGBoost) **B**, ROC curves for the optimized XGBoost model on internal test set (top) and external validation (bottom). **C**, Calibration analysis: Internal test set (top) and external validation (bottom). **D**, Decision curve analysis: Internal test set (top) and external validation (bottom). **E**, SHAP value visualizations showing feature importance for PARP inhibitor efficacy prediction (red = high feature values, blue = low values).

Building upon prior foundational research that elucidated the mechanisms underlying the enhanced efficacy of PARPi through Met in PTEN-deficient prostate cancer patients, we developed a predictive model to estimated patient response likelihood to PARPi. This study further investigated whether Met combination therapy could improve high-dose PARPi effectiveness. We generated 11 machine learning models to predict PSA50 response following Olaparib treatment. The XGBoost model demonstrated optimal predictive performance, achieving an AUC of 0.78 (Figure 9A). Given the retrospective study's limited sample size, we employed grid search and cross-validation to optimize the CatBoost model, which showed robust performance with an AUC of 0.8 in internal testing and 0.73 in external validation (Figure 9B). Additional evaluations, including calibration curves (0.88 for internal and 0.79 for external cohorts) and decision curve analysis (with threshold ranges of 0-80% and 0-70%, respectively) (Figures 9C, D), confirming strong discriminative power and clinical utility.

**Figure 10:**
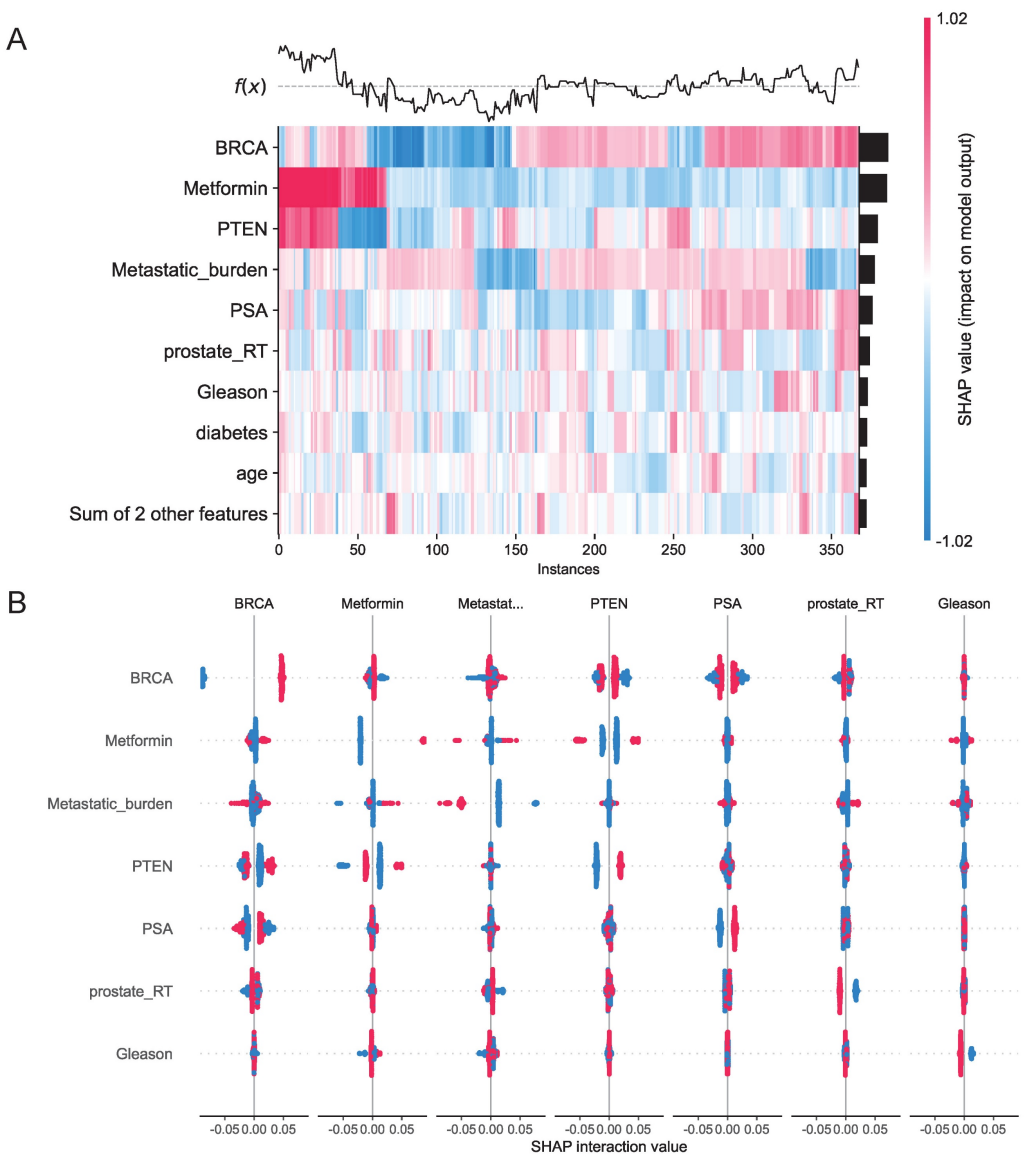
** A**, Interaction Summary Plot: A visualization method displaying feature interaction importance and impact. Features are ranked vertically by importance. Total importance for each feature equals the sum of its interactions with all other features. SHAP interaction values are represented by dots, where dot size corresponds to predictive influence strength. Color gradients represent interacting features. **B**, SHAP Heatmap: The left y-axis ranks features by importance (highest at top). The right y-axis visualizes feature impact, with darker colors indicating larger absolute SHAP values and greater predictive influence. The upper section displays model prediction outcomes.

We assessed 11 models using seven performance metrics (accuracy, sensitivity, specificity, precision, negative predictive value, F1-score, and Cohen's Kappa.) for both internal testing and external validation (Supplementary Figures 8A, B). Given that the XGBoost model is often considered a 'black box' due to its opaque internal mechanisms and non-transparent decision processes, this study employed SHAP values to systematically quantify each variable's contribution to the predictions, thereby enhancing the interpretability of the model's outputs. As illustrated in the SHAP summary plot (Figure 9E), the importance of each feature was ranked in descending order based on its SHAP value contribution. Following the SHAP analysis, we created an interaction summary plot (Figure 10A) to examine feature relationships. To enhance interpretability, we generated a SHAP heatmap (Figure 10B). We also created decision plots to identify features causing incorrect predictions. These plots helped refine the model and guided feature optimization for subsequent tuning (Supplementary Figure 8C).

To facilitate clinical implementation, we deployed the final predictive model as a web-based platform, as shown in Supplementary Figure 7. When users input values for the 11 required features, the program automatically predicts the patient's likelihood of responding to PARPi and displays the corresponding force plot and LIME analysis (Local Interpretable Model-agnostic Explanations) analysis, illustrating the proportional contribution of each feature to the prediction (Supplementary Figure 9). For Met-naïve patients, the platform predicts both PARP inhibitor monotherapy efficacy and the potential response improvement from Met combination therapy. This platform is accessible at https://jbzx4rotq9uedmghf4ctya.streamlit.app/.

## Discussion

In this study, we demonstrate that PTEN-deficient PCa develops resistance to PARPi in a lactate-enriched microenvironment and identify Met as a metabolic modulator capable of effectively reversing tumor cell resistance to PARP inhibition. Mechanistic investigations reveal that both lactate-induced resistance and Met-mediated sensitivity regulation operate through modulation of intracellular NADP^+^ levels. Further research demonstrated that the activation of NOX1, an NADP^+^-generating enzyme, is closely linked to the expression of PTEN and NHE1 proteins. Notably, our transcriptomic analysis revealed that PTEN loss leads to significant upregulation of key glycolytic genes, thereby promoting a metabolic shift toward glycolysis and enhancing lactate production. Thus, while PTEN loss confers PARPi resistance to tumor cells, this molecular vulnerability simultaneously establishes the foundation for Met to enhance the antitumor efficacy of PARPi.

According to Hanahan's redefined hallmarks of cancer, genomic instability and dysregulated cellular energetics are established as two fundamental features of malignancy 49. Notably, PARP enzyme activity is directly modulated by cellular NAD^+^ availability 50, 51. PARP activation consumes NAD^+^ to synthesize poly (ADP-ribose), depleting intracellular NAD^+^ by 30-80% during DNA damage. This effect is particularly severe in DNA repair-deficient cells 52. Given that PARPi are primarily used to treat tumors with HR deficiency, these cancer cells transition from glycolytic metabolism (Warburg effect) toward oxidative metabolism, primarily through enhanced OXPHOS activity 19, thereby substantially replenishing NAD^+^ to support DNA repair. Since DNA damage response is intrinsically linked to cellular metabolism 20-22, HR-deficient tumor cells consequently exhibit a weakened Warburg phenotype, leading to reduced extracellular lactate levels and reduced PARPi resistance, which explains their therapeutic vulnerability to these inhibitors.

Previous studies have shown that high glycolytic flux reduces tumor cell sensitivity to olaparib 19, a finding consistent with our own results. We further demonstrate that glycolysis-driven increases in intracellular NADP⁺ levels contribute to resistance against PARPi therapy. PTEN, among the most frequently altered tumor suppressors, serves as a central regulator of cellular metabolism. As reported by Isabel Garcia-Cao et al., high PTEN expression promotes oxidative phosphorylation while suppressing the Warburg effect and preserving insulin sensitivity 53. Conversely, PTEN deficiency enhances glycolytic metabolism (Warburg effect) and is associated with insulin resistance and hyperglycemia 54. These findings collectively establish that PTEN modulates tumor metabolism in a way that influences responsiveness to PARPi. Metformin, as a widely used metabolic modulator, may thereby further modify PARPi efficacy within this metabolic context. It should be noted that PTEN regulates metabolism through multiple mechanisms, both in the cytoplasm and nucleus, suggesting that additional pathways beyond those discussed here may also contribute to PARPi sensitivity. Further investigation will be required to fully elucidate these potential mechanisms.

Notably, a recent Nature study suggests that the antitumor effects of PARPi remain controversial 57. The study showed that PARPi's synthetic lethality occurs through blocking transcription-replication conflict signals, not PARP trapping. This blockade subsequently induces DNA damage. Additionally, Met-induced cell cycle arrest may also modulate the efficacy of PARP inhibitors 58, providing new insights into the interaction between Met and PARP inhibitors. However, as the multiple mechanisms underlying PARP inhibitors' DNA repair suppression remain under debate, the antitumor mechanism of PARPi driven by significantly elevated NADP^+^ levels in tumor cells may represent a distinct mechanism that warrants further investigation.

Our animal experiments and clinical analyses show that Met enhances PARP inhibitors' antitumor effects in PTEN-deficient prostate tumors, though some mechanisms require further study. Building upon these findings, we developed and validated a predictive model for PARPi efficacy using comprehensive clinical data. To our knowledge, this represents the first predictive model specifically designed to evaluate PARP inhibitor efficacy in prostate cancer patients. Because the factors interact complexly, we used several machine learning methods to better understand these relationships and improve prediction accuracy. However, the inherent opacity of machine learning analyses may raise concerns among clinicians. To address this limitation, we implemented the SHAP method to enhance model interpretability. The SHAP method offers both broad understanding of the model's function and specific insights for individual patients, showing how personalized data generates each prediction. We therefore created a web-based platform using Streamlit to make the model easily accessible to clinicians.

Nevertheless, this study has several limitations: 1. As a retrospective analysis, possible selection bias exists, necessitating validation through prospective studies. 2. The small sample size and limited study duration resulted in elevated survival rates in OS analysis, making it difficult to accurately assess the impact of PARP inhibitors on OS. 3. Some BRCA-mutated patients exhibited poor responses to PARP inhibitors. Future studies with larger sample sizes could identify factors contributing to enhanced glycolytic phenotypes and drug resistance. 4. the current model has not yet incorporated comprehensive analyses of other potential predictors. Integration of additional multi-omics data, such as radiomics and pathomics profiles, could significantly improve the model's accuracy and explanatory power. In future research, our team plans to investigate the influence of NADP^+^ on PARP protein structural isomerization and the role of metabolism in drug resistance among BRCA-mutated patients.

In summary, our findings demonstrate that the lactate microenvironment can induce PARPi resistance in PTEN-deficient prostate cancer, while Met application effectively reverses this phenomenon, providing robust theoretical support for overcoming clinical limitations in PARPi therapy.

## Supplementary Material

Supplementary figures and tables.

## Abbreviations

PCa: Prostate cancer
CRPC: Castration-resistant prostate cancer
mCRPC: Metastatic castration-resistant prostate cancer
HRR: Homologous Recombination Repair
PFS: Progression-Free Survival
OS: Overall survival
PARP: Poly (ADP-ribose) polymerase
PARPi: Poly (ADP-ribose) polymerase inhibitors
Met: Metformin
PSA: Prostate-specific antigen
SHAP: SHapley Additive exPlanations
LIME: Local Interpretable Model-agnostic Explanations
IC50: The half-maximal inhibitory concentration
3D: Three-dimensional
ROS: Reactive oxygen species
OXPHOS: oxidative phosphorylation
LA: lactic acid
HA: hydrochloric acid
SL: Sodium lactate
NAC: N-Acetyl-L-cysteine
NOX: NADPH oxidase
APO: Apocynin
CRD: Cariporide
NHE1: Na^+^/H^+^ exchanger 1
CCK8: Cell Counting Kit-8
WB: Western blot
ROC: Receiver operating characteristic
AUC: Area under curve
