# Supplementary material for: Metformin Sensitizes PTEN-deficient Prostate Cancer to PARP Inhibitors by Rebuilding NADP+ Homeostasis
Source: Int J Biol Sci. 2026 Jan 8;22(3):1346–68. doi: 10.7150/ijbs.121033 (PMC12837654; doi:10.7150/ijbs.121033)
Supplement: Supplementary file 1 — Supplementary figures and tables. [file ijbsv22p1346s1.pdf]

Supplementary data

Supplementary Figure 1 **A**, **B**, Survival analysis grouped by Met treatment, with corresponding PFS and OS curves. **C**, **D**, Subgroup analysis of Met-treated patients stratified by PTEN mutation status, with corresponding PFS and OS curves. **E**, **F**, Patients stratified by PTEN status (positive/negative) and Met treatment (yes/no) into four groups, with corresponding PFS and OS curves.

Supplementary Figure 2: **A**, CCK-8 assay measured the proliferative activity of PC3 and DU145 cells in Control, HA, SL, PARPi, HA+PARPi, and SL+PARPi groups (top panel). The middle and bottom panels show the proliferation of LNCaP and 22Rv1 cells in Control, LA, HA, SL, PARPi, and their respective combination groups (LA+PARPi, SL+PARPi, and HA+PARPi). **B**, Colony formation assay was used to assess the clonogenic efficiency of PC3 and DU145 cells in SL, HA, and their combination with PARPi groups. The right panel shows the quantitative analysis of colony counts. **C**, After 48 hours of treatment with SL, HA, SL+PARPi, and HA+PARPi, apoptosis in PC3 and DU145 cells was detected via flow cytometry. The right panel provides quantitative data on apoptotic cells. **D**, CCK-8 assay evaluated the proliferation of four prostate cancer cell lines (PC3, LNCaP, DU145, and 22Rv1) under the following conditions: (i) control (untreated); (ii) LA medium alone; and (iii) LA medium containing three different PARP inhibitors (alazoparib, rucaparib, and olaparib).

Supplementary Fig. 3 **A,** Volcano plot analysis of differentially expressed genes from RNA-seq after PTEN-knockdown in DU145 cells. **B**,**C**. Gene set enrichment analysis (GSEA) demonstrating significant upregulation of the "lactate metabolic process" gene set in DU145-PTEN-KD cells (normalized enrichment score (NES) = 1.43; P = 0.04). **D**. Gene Ontology (GO) molecular function enrichment analysis of DEGs. Top significantly upregulated GO terms include NAD^+ nucleosidase activity and $NAD(P)^+$ nucleosidase activity.

Supplementary Fig. 4 **A**, Measurement of NAD^+ and $NADP^+$ concentrations in PC3, DU145, PC3-PTEN-OE, and DU145-PTEN-KD tumor spheroids under different culture conditions. **B**, Imaging of ROS production by confocal microscopy in PC3 and DU145 cells under Control, LA, Met, and LA+Met conditions (left), with quantitative ROS level analysis (right). Scale bar: 100 μm. **C**, Confocal microscopy images showing ROS production in PC3 and DU145 cells under Control, LA, LA+NAC, and ROS conditions (left), with quantitative ROS level analysis (right). Scale bar: 100 μm. **D**, CCK-8 assay evaluating proliferation of PC3 and DU145 cells in two treatment groups: Group 1 - Baseline treatments: Control, LA, ROS (2 mM), ROS (4 mM), and LA+NAC; (2) Mefuparib-treated groups (same as baseline with 2 μM mefuparib). **E**, Flow cytometry analysis of apoptosis in PC3 and DU145 cells after 48 h treatment with Control, PARPi, PARPi+ROS, LA+PARPi+NAC, PARPi+$NADP^+$ (30 mM), and PARPi+$NADP^+$ (100 mM). Quantitative apoptosis data are shown (right). **F**, Comet assay assessing DNA damage in PC3 and DU145 cells under Control, PARPi, PARPi+ROS, LA+PARPi+NAC, PARPi+$NADP^+$ (30 mM), and PARPi+$NADP^+$ (100 mM) conditions. Quantitative DNA damage analysis is shown (right). Scale bar: 1 μm.

Supplementary Figure 5：**A**, Confocal microscopy images showing ROS levels in DU145 cells under Control, LA, ROS, and LA+ROS conditions (left), with quantitative analyses of intracellular ROS (middle) and $NADP^+$ levels (right). **B**, Western blot (WB) analysis of PTEN, NOX, p-NOX, and β-actin protein levels in DU145 cells under Control, LA, ROS, and LA+ROS conditions, with

band density quantification (right). **C**, Comet assay assessing DNA damage in DU145 cells under Control, PARPi, LA+PARPi, and LA+PARPi+ROS conditions, with DNA damage quantification (bottom). Scale bar: 1 μm. **D**, Flow cytometric analysis of apoptosis in DU145 cells treated under Control, PARPi, LA+PARPi, and LA+PARPi+ROS conditions for 48 hours (bottom: quantitative apoptosis analysis). **E**, DU145 cells cultured in ultra-low-attachment 96-well plates for 48 hours, then treated with Control, PARPi, or PARPi+ROS media for 72 hours. Fluorescence images show calcein-AM (green, live cells) and PI (red, dead cells) staining. Tumor spheroid fluorescence ratios were quantified using ImageJ (right). Scale bar: 500 μm. **F**, Confocal microscopy images of ROS secretion in PC3-PTEN-OE and DU145-PTEN-KD cells under Control, LA, LA+ROS, and LA+Met conditions (left), with quantitative analyses of ROS (middle) and $NADP^+$ secretion levels (right).

Supplementary Figure 6**A**, Colony formation assays were used to assess the colony-forming capacity of PC3 cells in the Control, PARPi, LA+PARPi, and LA+PARPi+APO groups, with quantitative results shown adjacent. **B**, CCK8 assays were used to quantify proliferative activity of DU145 cells in the Control, CRD, PARPi, LA+PARPi, and LA+PARPi+CRD groups. **C**, CCK8 assays were used to determine the IC50 values of PARPi in DU145 cells treated with LA or LA+CRD. **D**, Western blot (WB) analysis measured NHE1 and β-actin protein levels in PC3, DU145, PC3-NHE1-KD, and DU145-NHE1-KD cells, with band density quantification shown beneath. **E**, NHE1 activity was evaluated using the NH4Cl pulse assay. The time course of pHi recovery from cytoplasmic acid load to baseline was measured in PC3 and DU145 cells with or without NHE1 inhibitor, as well as in PC3-NHE1-KD and DU145-NHE1-KD cells. **F**, PC3, DU145, and PC3-NHE1-KD cells were treated in different media for 48 hours, and apoptosis levels were determined by flow cytometry. The right panel shows quantitative analysis of apoptotic cells. **G**, CCK8 assays were used to quantify the proliferation of PC3-NHE1-KD cells in the Control, PARPi, LA, and LA+PARPi groups. **H**, DNA damage in PC3 and DU145 cells from the Control, PARPi, LA+PARPi, and LA+PARPi+CRD groups was measured by comet assay, with quantitative results shown adjacent. **I**, Colony formation assays were used to evaluate the colony-forming capacity of PC3 and DU145 cells in the Control, PARPi, LA+PARPi, and LA+PARPi+CRD groups, with quantitative results shown adjacent. **J**, DNA damage and colony-forming capacity of PC3-NHE1-KD cells in the Control, PARPi, and LA+PARPi groups were assessed by comet assay and colony formation assay, respectively, with quantitative results shown adjacent.

Supplementary Figure 7. **A,** Molecular docking models depicting the binding interactions of Talazoparib, Olaparib, Niraparib, and $NADP^+$ within the catalytic site of PARP. **B,** Proliferation of PC3 and DU145 cells was measured by CCK-8 assay across four treatment conditions: Control, PARPi alone, PARPi + 30 μM $NADP^+$, and PARPi + 120 μM $NADP^+$. Experiments were performed using three distinct PARP inhibitors: Talazoparib, Olaparib, and Niraparib. **C,** Clonogenic survival of PC3 and DU145 cells treated with the same three PARP inhibitors under the four experimental groups described in panel B. Colony formation was evaluated at $NADP^+$ concentrations of 30 μM and 120 μM. **D,** Apoptosis was assessed by flow cytometry in PC3 and DU145 cells after 48 hours of treatment according to the schemes outlined in panel B. The right panel shows quantitative analysis of apoptotic cells. Supplementary Figure 8. **A**, Performance metrics including accuracy, sensitivity, specificity, precision, negative predictive value, F1-score, and Cohen's kappa score for seven prediction models (Decision Tree, SVM, KNN, Logistic Regression, GNB, MLP, and Random Forest) in the internal test set (top) and external validation set (bottom). **B**, The same

metrics for four boosting ensemble models (LightGBM, XGBoost, CatBoost, and NGBoost) in the internal test set (top) and external validation set (bottom). **C**, Decision plot visualizing misclassified samples based on SHAP explanation values. By computing SHAP values for model predictions and combining them with the expected output value, this plot highlights feature contributions and misclassified samples. Blue and red lines indicate negative and positive contributions to predictions, respectively, helping identify influential features for model refinement.

Supplementary Figure 9. The final XGBoost model with 11 features predicts PARPi efficacy. Users can input actual feature values to generate PARPi efficacy probabilities. The force plot shows feature contributions to PARPi efficacy predictions: blue bars (right side) indicate features pushing toward 'ineffective' predictions, while red bars (left side) suggest 'effective' predictions. LIME bar charts similarly show feature contributions, with blue representing 'ineffective' and orange indicating 'effective' predictions. The accompanying table shows the sample's actual feature values, enabling users to verify which factors drove the model's prediction.

Table1：Comparison of patient baseline characteristics in the development cohort

Table2：Multivariate logistic regression for predicting PFS and OS.

Table3：Univariate and multivariate logistic regression for predicting PSA50

FigS1

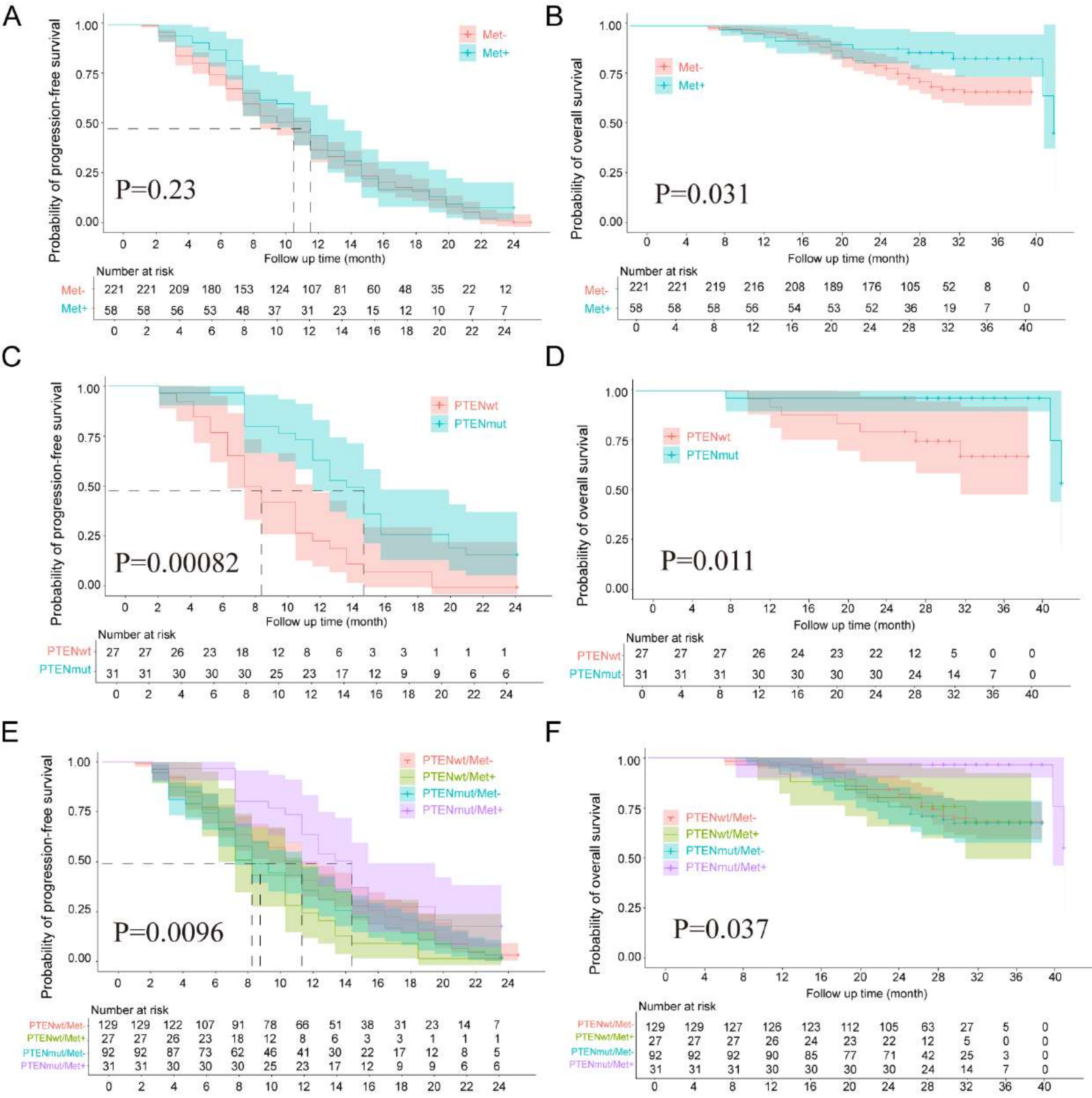
A
Probability of progression-free survival
Met-
Met+
P=0.23
Follow up time (month)
Number at risk
Met- 221 221 209 180 153 124 107 81 60 48 35 22 12
Met+ 58 58 56 53 48 37 31 23 15 12 10 7 7
B
Probability of overall survival
Met-
Met+
P=0.031
Follow up time (month)
Number at risk
Met- 221 221 219 216 208 189 176 105 52 8 0
Met+ 58 58 58 56 54 53 52 36 19 7 0
C
Probability of progression-free survival
PTENwt
PTENmut
P=0.00082
Follow up time (month)
Number at risk
PTENwt 27 27 26 23 18 12 8 6 3 3 1 1 1
PTENmut 31 31 30 30 30 25 23 17 12 9 9 6 6
D
Probability of overall survival
PTENwt
PTENmut
P=0.011
Follow up time (month)
Number at risk
PTENwt 27 27 27 26 24 23 22 12 5 0 0
PTENmut 31 31 31 30 30 30 30 24 14 7 0
E
Probability of progression-free survival
PTENwt/Met-
PTENwt/Met+
PTENmut/Met-
PTENmut/Met+
P=0.0096
Number at risk
PTENwt/Met- 129 129 122 107 91 78 66 51 38 31 23 14 7
PTENwt/Met+ 27 27 26 23 18 12 8 6 3 3 1 1 1
PTENmut/Met- 92 92 87 73 62 46 41 30 22 17 12 8 5
PTENmut/Met+ 31 31 30 30 30 25 23 17 12 9 9 6 6
F
Probability of overall survival
PTENwt/Met-
PTENwt/Met+
PTENmut/Met-
PTENmut/Met+
P=0.037
Follow up time (month)
Number at risk
PTENwt/Met- 129 129 127 126 123 112 105 63 27 5 0
PTENwt/Met+ 27 27 27 26 24 23 22 12 5 0 0
PTENmut/Met- 92 92 92 90 85 77 71 42 25 3 0
PTENmut/Met+ 31 31 31 30 30 30 30 24 14 7 0

FigS2

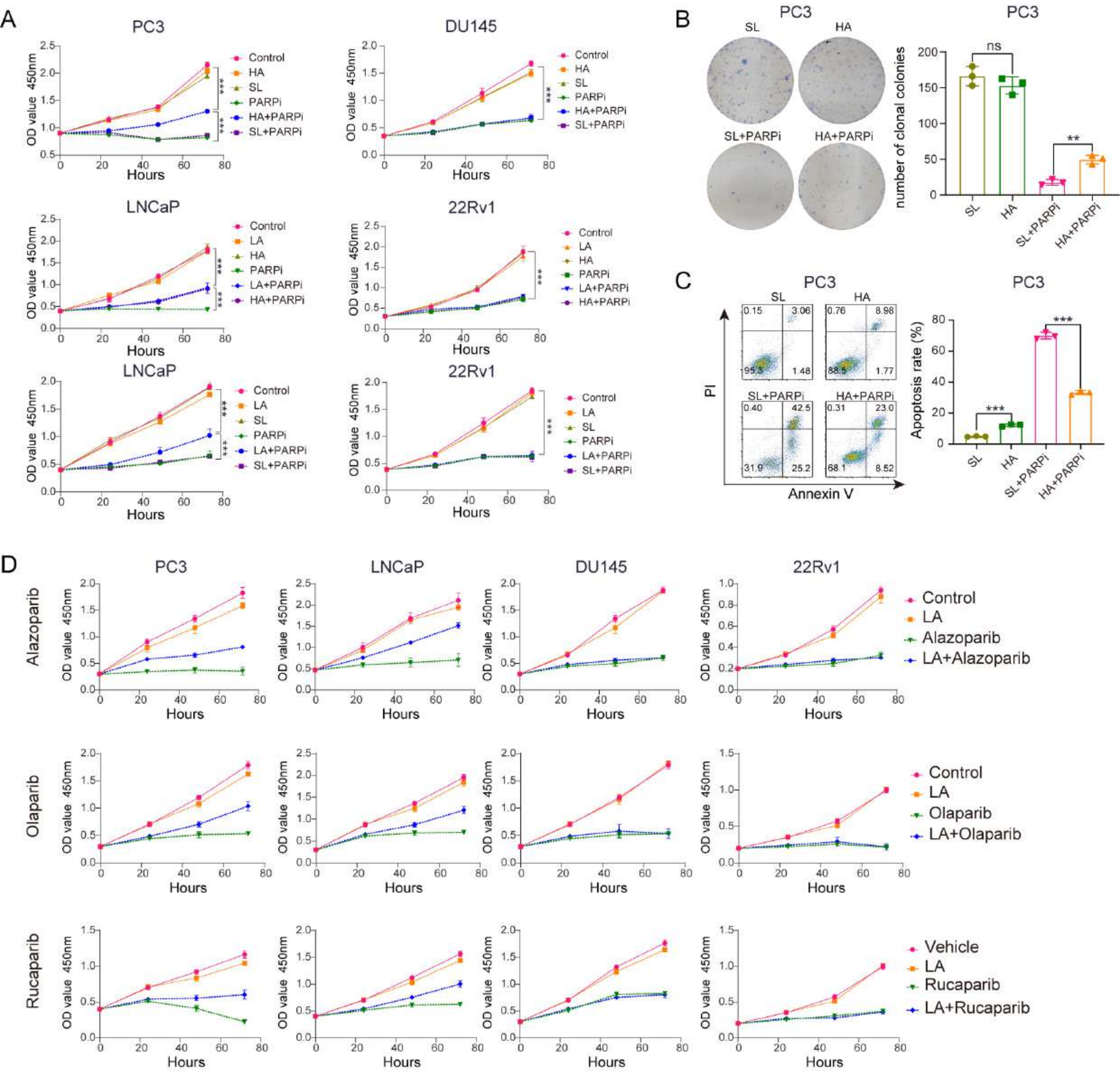
A
PC3
DU145
LNCaP
22Rv1
OD value 450nm
Hours
Control
HA
SL
LA
PARPi
HA+PARPi
SL+PARPi
LA+PARPi
***
B
PC3
SL
HA
SL+PARPi
HA+PARPi
number of clonal colonies
ns
**
C
PC3
PI
Annexin V
0.15
3.06
95.3
1.48
0.76
8.98
88.5
1.77
0.40
42.5
31.9
25.2
0.31
23.0
68.1
8.52
Apoptosis rate (%)
***
D
PC3
LNCaP
DU145
22Rv1
Alazoparib
Olaparib
Rucaparib
Control
Vehicle
LA
LA+Alazoparib
LA+Olaparib
Rucaparib
LA+Rucaparib

FigS3

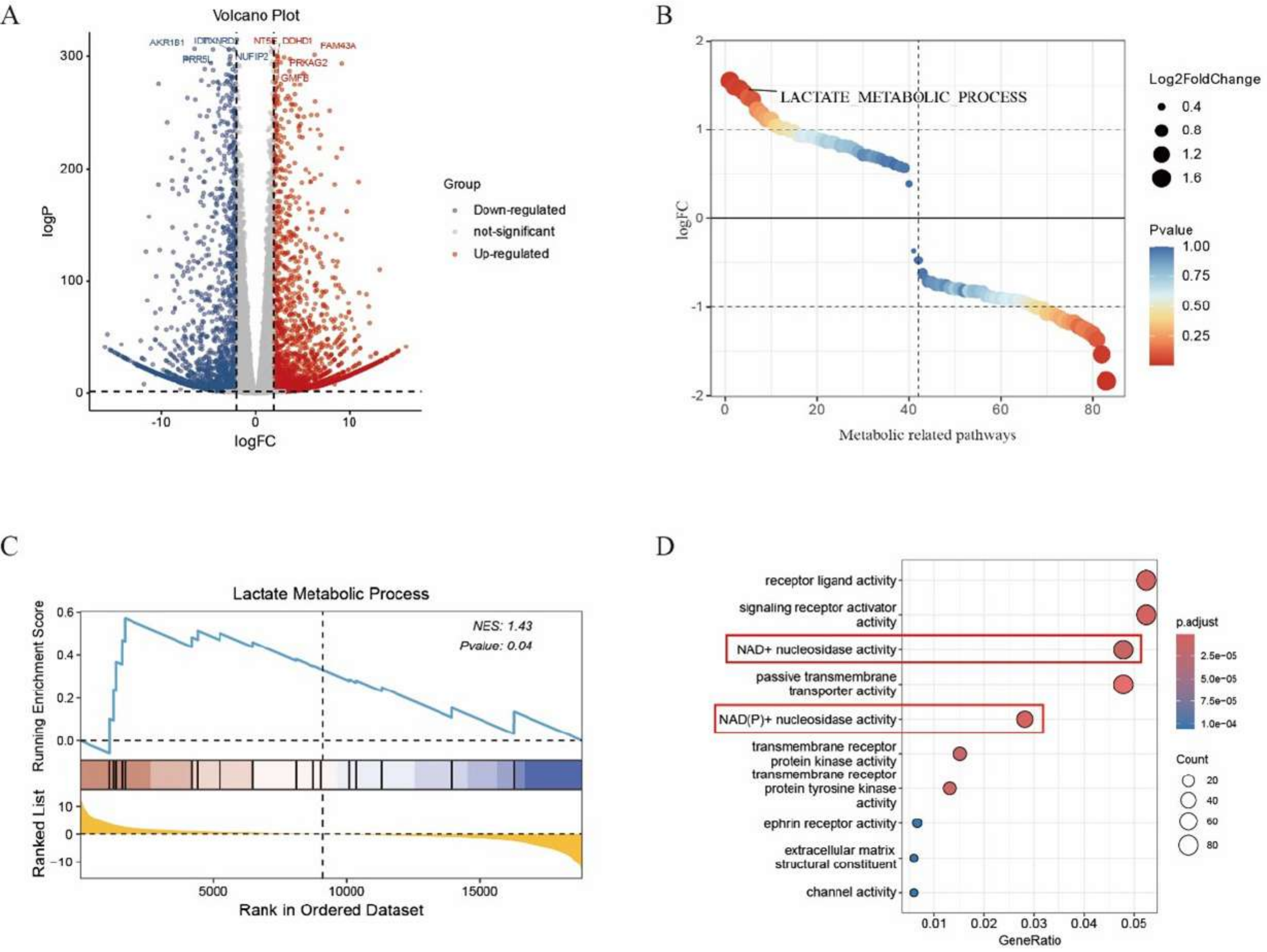
A
Volcano Plot
AKR1B1
PRR5L
NUFIP2
NT5E
DDHD1
FAM43A
PRKAG2
GMFB
logP
logFC
Group
Down-regulated
not-significant
Up-regulated
B
LACTATE_METABOLIC_PROCESS
logFC
Metabolic related pathways
Log2FoldChange
Pvalue
C
Lactate Metabolic Process
NES: 1.43
Pvalue: 0.04
Running Enrichment Score
Ranked List
Rank in Ordered Dataset
D
receptor ligand activity
signaling receptor activator activity
NAD+ nucleosidase activity
passive transmembrane transporter activity
NAD(P)+ nucleosidase activity
transmembrane receptor protein kinase activity
transmembrane receptor protein tyrosine kinase activity
ephrin receptor activity
extracellular matrix structural constituent
channel activity
GeneRatio
p.adjust
Count

FigS4

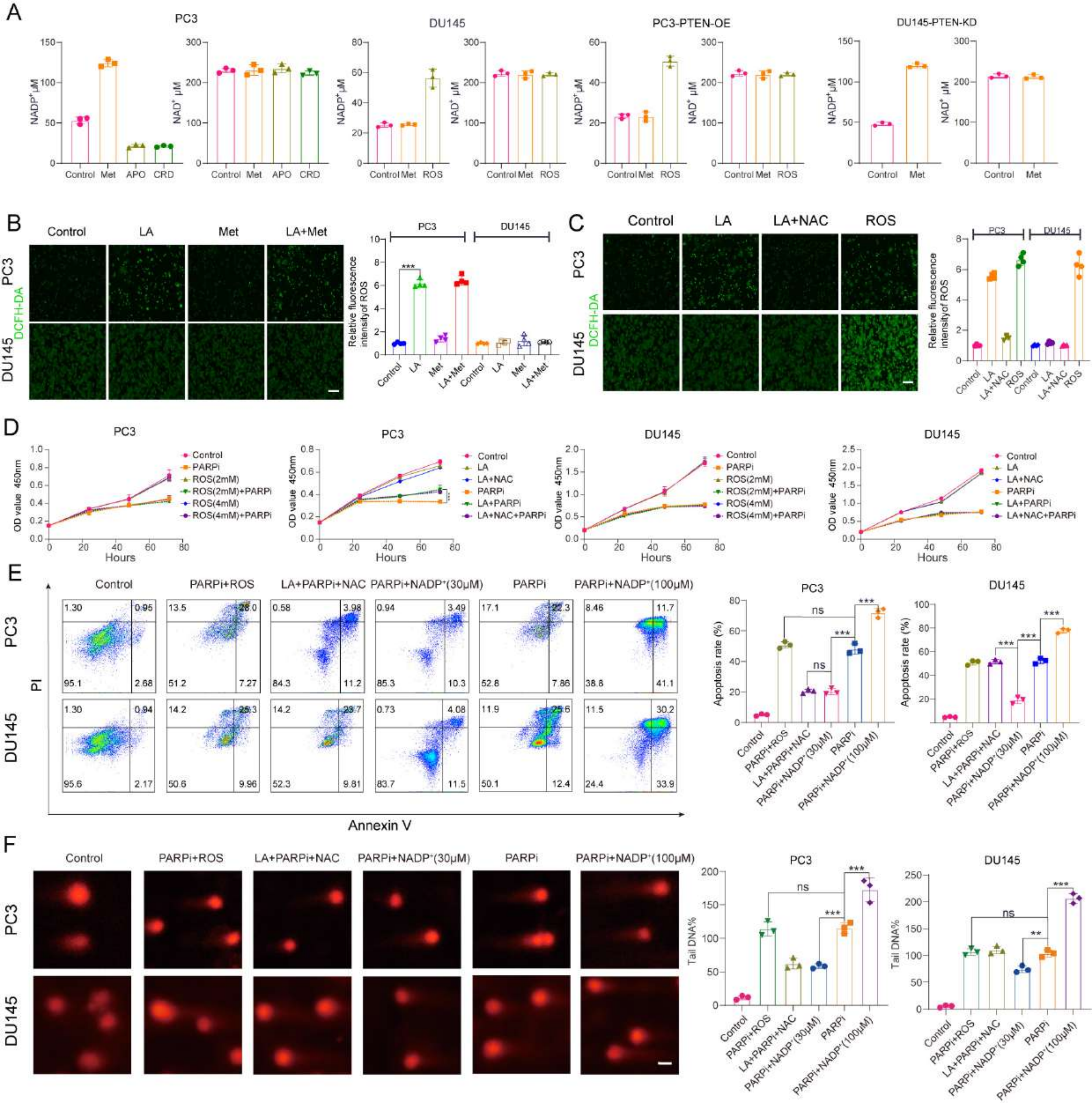
A
PC3
DU145
PC3-PTEN-OE
DU145-PTEN-KD
NADP⁺ μM
NAD⁺ μM
Control
Met
APO
CRD
ROS
B
Control
LA
Met
LA+Met
PC3
DU145
DCFH-DA
Relative fluorescence intensityof ROS
C
LA+NAC
ROS
D
OD value 450nm
Hours
PARPi
ROS(2mM)
ROS(2mM)+PARPi
ROS(4mM)
ROS(4mM)+PARPi
LA+PARPi
LA+NAC+PARPi
E
PARPi+ROS
LA+PARPi+NAC
PARPi+NADP⁺(30μM)
PARPi+NADP⁺(100μM)
PI
Annexin V
Apoptosis rate (%)
ns
F
Tail DNA%

FigS5

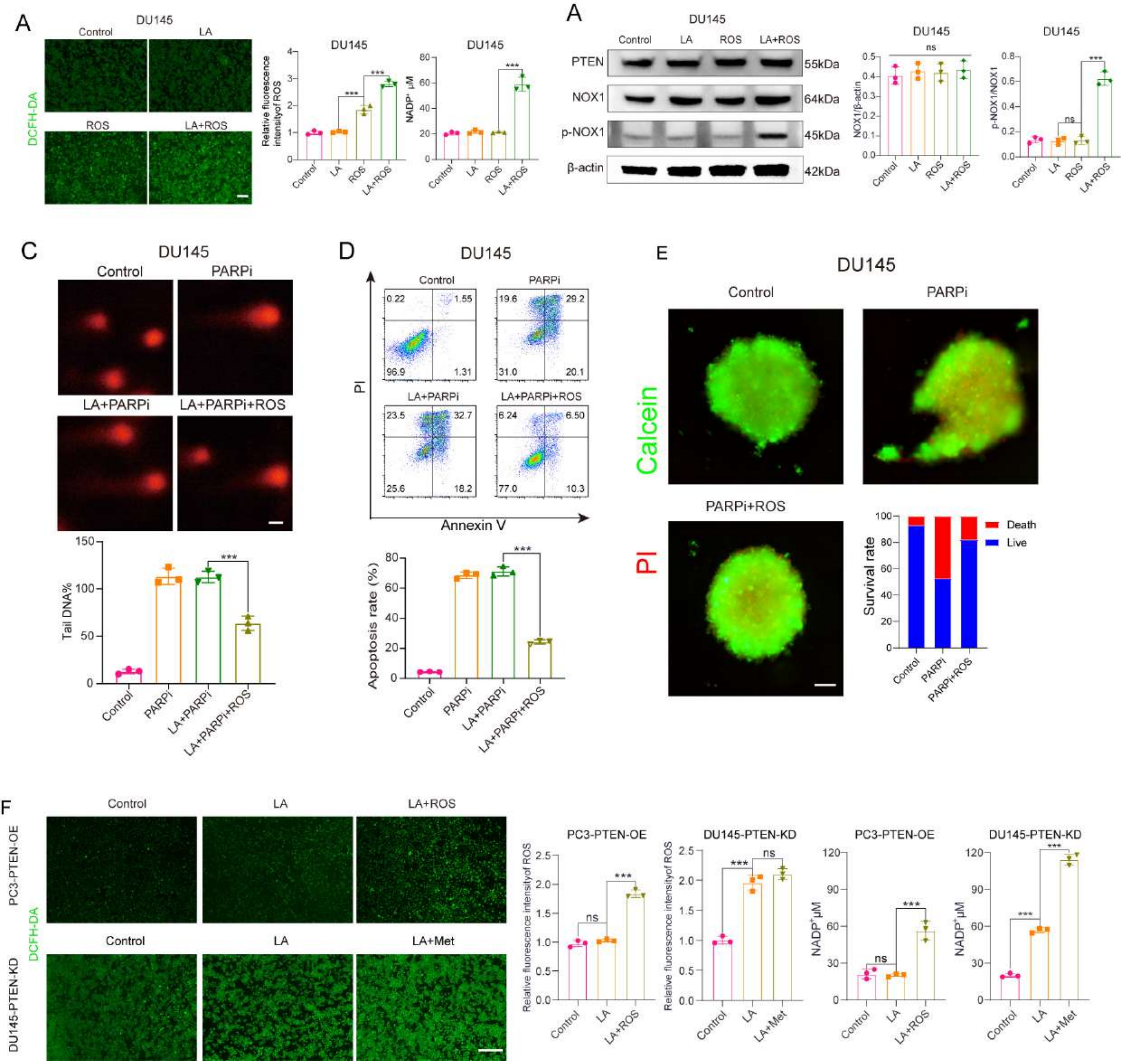
A
DU145
Control
LA
ROS
LA+ROS
DCFH-DA
Relative fluorescence intensityof ROS
NADP+ μM
Control
LA
ROS
LA+ROS
PTEN
NOX1
p-NOX1
β-actin
55kDa
64kDa
45kDa
42kDa
NOX1/β-actin
p-NOX1/NOX1
ns
***
C
PARPi
LA+PARPi
LA+PARPi+ROS
Tail DNA%
D
PI
Annexin V
0.22
1.55
96.9
1.31
19.6
29.2
31.0
20.1
23.5
32.7
25.6
18.2
6.24
6.50
77.0
10.3
Apoptosis rate (%)
E
PARPi+ROS
Calcein
PI
Death
Live
Survival rate
F
PC3-PTEN-OE
DU145-PTEN-KD
LA+Met
Relative fluorescence intensityof ROS
NADP+ μM

FigS6

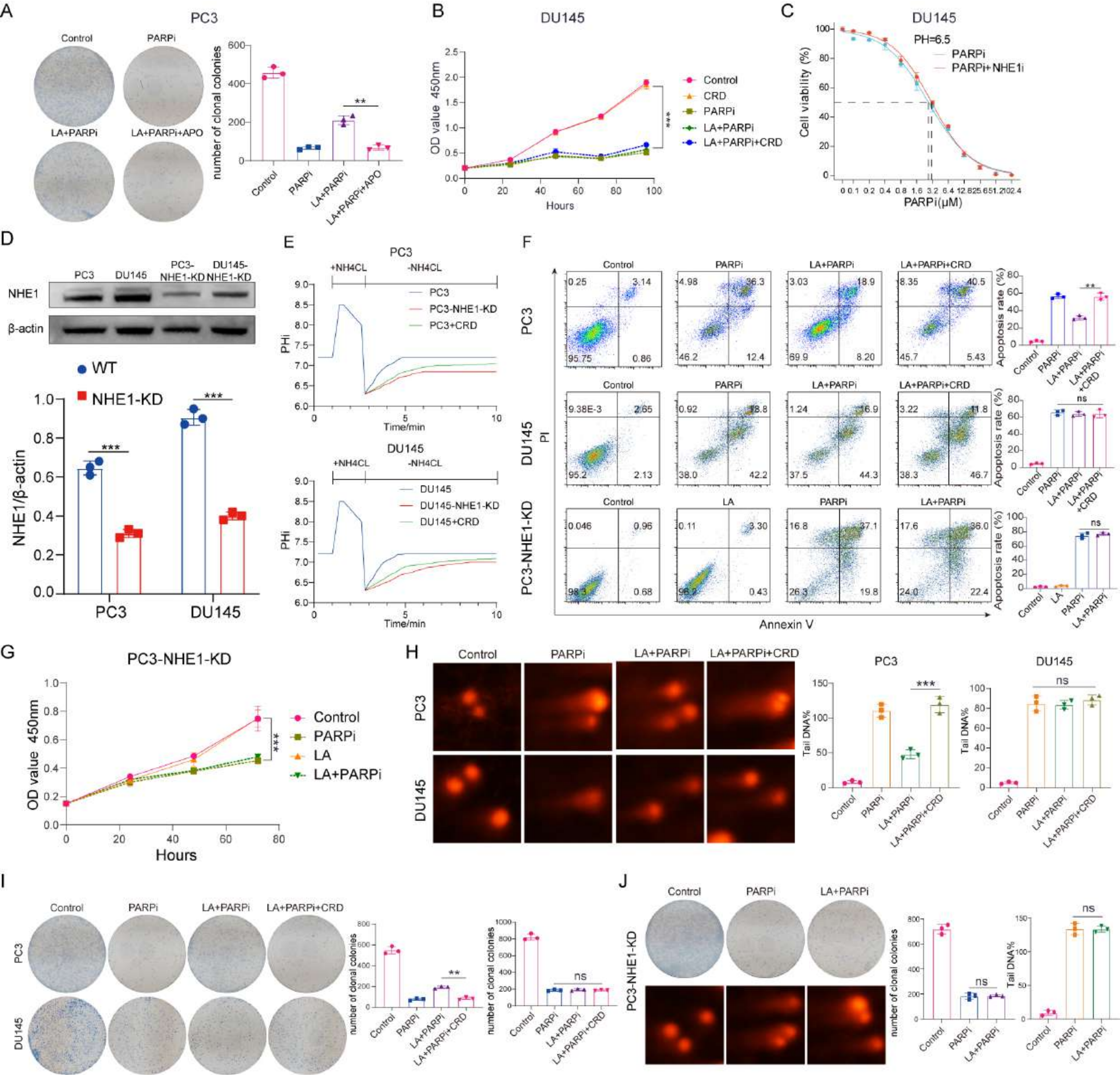
A
PC3
Control
PARPi
LA+PARPi
LA+PARPi+APO
number of clonal colonies
B
DU145
OD value 450nm
Hours
Control
CRD
PARPi
LA+PARPi
LA+PARPi+CRD
C
DU145
PH=6.5
PARPi
PARPi+NHE1i
Cell viability (%)
PARPi(μM)
D
PC3
DU145
PC3-NHE1-KD
DU145-NHE1-KD
NHE1
β-actin
WT
NHE1-KD
NHE1/β-actin
E
+NH4CL
-NH4CL
PC3-NHE1-KD
PC3+CRD
DU145-NHE1-KD
DU145+CRD
PHi
Time/min
F
PI
Annexin V
Apoptosis rate (%)
LA
G
PC3-NHE1-KD
H
LA+PARPi+CRD
Tail DNA%
ns
I
J

FigS7

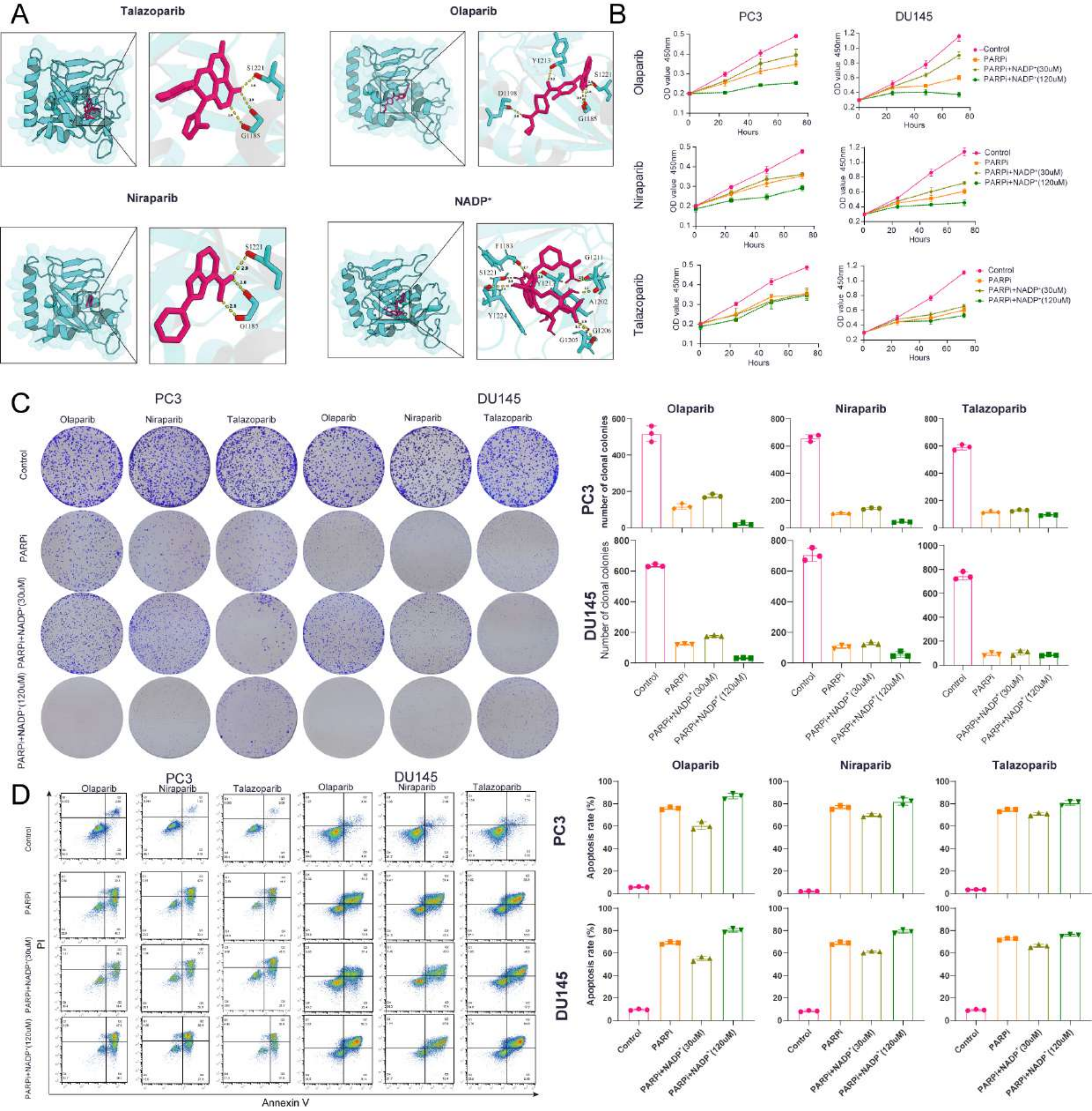
A
Talazoparib
Olaparib
Niraparib
NADP+
B
PC3
DU145
Olaparib
Niraparib
Talazoparib
OD value 450nm
Hours
Control
PARPi
PARPi+NADP+(30uM)
PARPi+NADP+(120uM)
C
PC3
DU145
Control
PARPi
PARPi+NADP+(30uM)
PARPi+NADP+(120uM)
number of clonal colonies
D
PI
Annexin V
Apoptosis rate (%)

FigS8

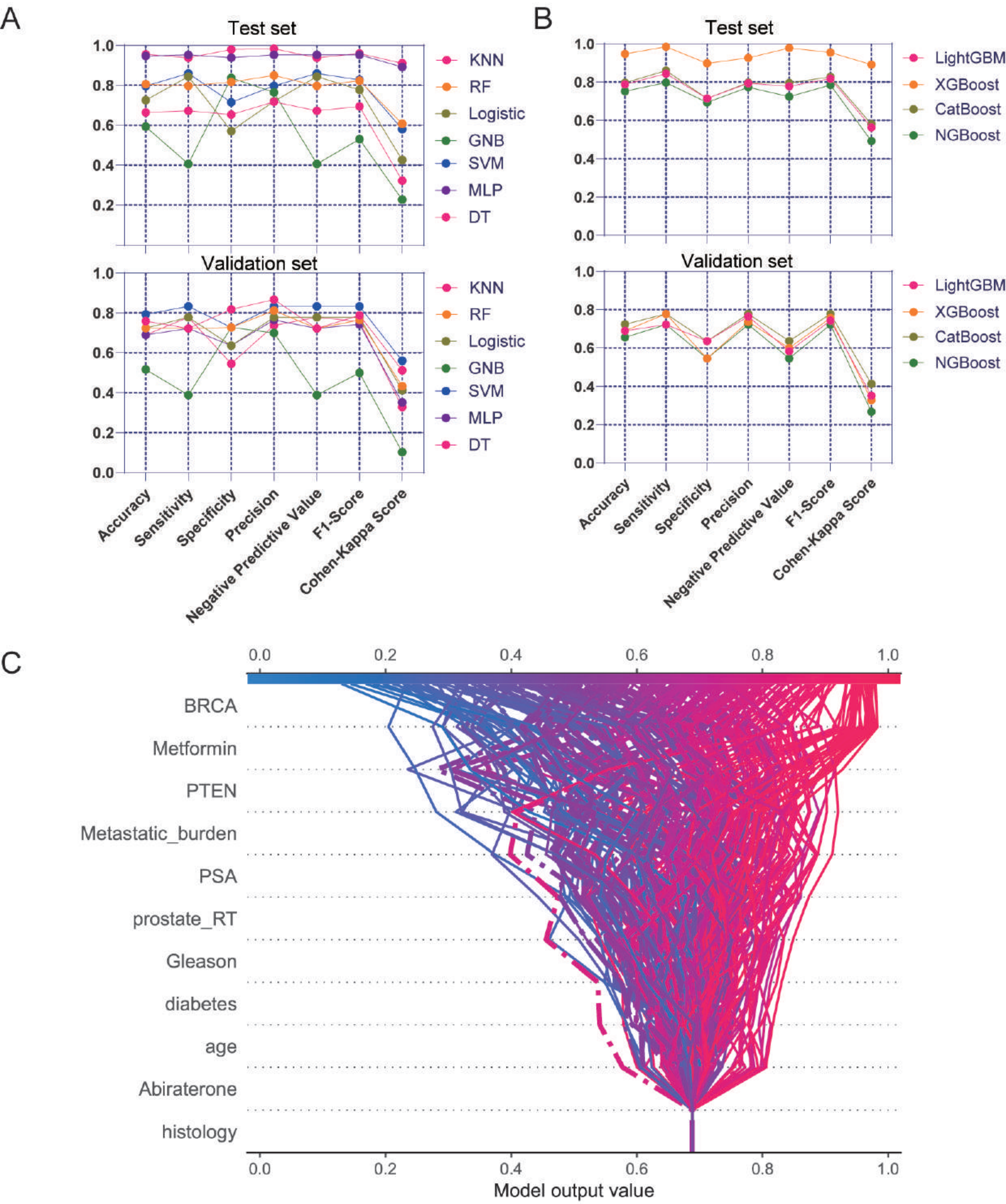
A
Test set
Validation set
1.0
0.8
0.6
0.4
0.2
0.0
KNN
RF
Logistic
GNB
SVM
MLP
DT
Accuracy
Sensitivity
Specificity
Precision
Negative Predictive Value
F1-Score
Cohen-Kappa Score
B
Test set
Validation set
LightGBM
XGBoost
CatBoost
NGBoost
C
BRCA
Metformin
PTEN
Metastatic_burden
PSA
prostate_RT
Gleason
diabetes
age
Abiraterone
histology
0.0
0.2
0.4
0.6
0.8
1.0
Model output value

FigS9

Predicted Class: 1 (1: Parp inhibitors are effective, 0: Parp inhibitors are ineffective)

Prediction Probabilities:[0.28085184 0.71914816]

According to our model, Parp inhibitors are effective. The probability of PARP inhibitors being effective is71.9%.

SHAP Force Plot Explanation

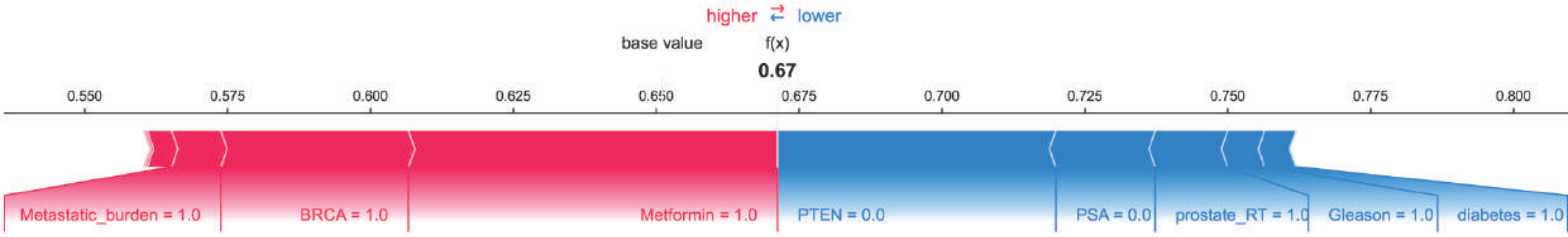

LIME Explanation

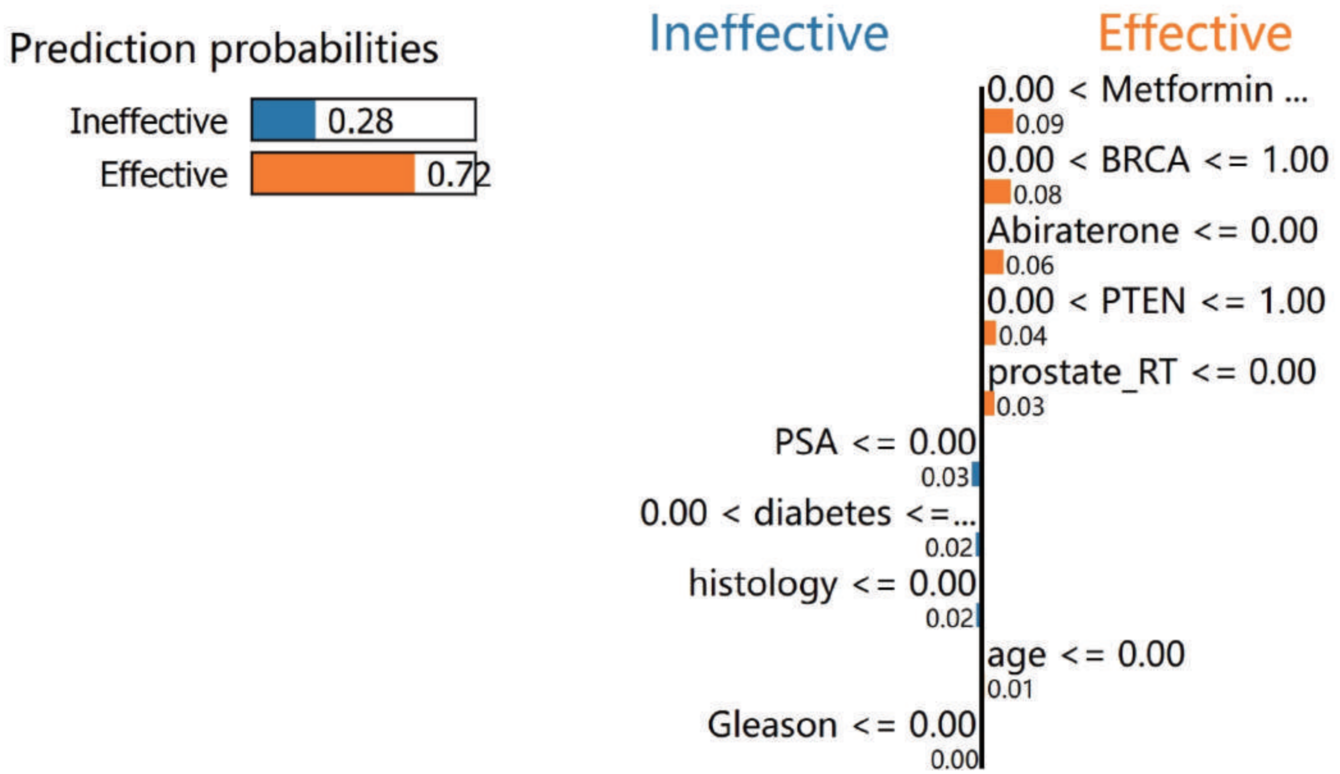

Table 1: Comparison of patient baseline characteristics in the development cohort

	Overall	Others	PTEN+/metformin+	P value
No. of patients	461	411	50	
Age(mean±SD,years)	70.2±8.4	70.1±8.5	70.5±7.8	0.734
PSA(mean±SD, ng/ml)	64.2±32.1	64±32.1	65.5±32.3	0.759
Histology, n (%)				0.651
Adenocarcinoma	447(97)	398(96.8)	49(98)	
Other	14(3)	13(3.2)	1 (2)	
Gleason score, n (%)				0.966
6-8	158(34.3)	141(34.3)	17(34)	
9-10	303(65.7)	270(65.7)	33(66)	
M stage, n (%)				0.359
M0	21(4.6)	20(4.9)	1(2)	
M1	440(95.4)	391 (95.1)	49(98)	
Prior prostate RT and RP, n (%)				0.554
Yes	185(40.1)	163(39.7)	22(44)	
No	276(59.9)	248(60.3)	28(56)	
Prior therapy at mCRPC stage, n (%)				0.409
Abiraterone	362(78.5)	325(79.1)	37(74)	
Others	99(21.5)	86(20.9)	13(26)	
PSA 50(mean±SD,%)	-48.8±43	-46.2±43.4	-70.4±32.2	<0.001
>-50%	168(36.4)	162(39.4)	6(12)	<0.001
≤-50%	293(63.6)	249(60.6)	44(88)	
Diabetes, n (%)				<0.001
No	284(61.6)	284(69.1)	0(0)	
Yes	177(38.4)	127(30.9)	50 (100)	
Metformin, n (%)				<0.001
No	362(78.5)	362(88.1)	0(0)	
Yes	99(21.5)	49(11.9)	50(100)	
PTEN alteration, n (%)				<0.001
No	266(57.7)	266(64.5)	0(0)	
Yes	195(42.3)	146(35.5)	50(100)	
BRCA alteration, n (%)				0.543
No	156(33.8)	141(34.3)	15(33.8)	
Yes	305(66.2)	270(65.7)	35(70)	
PFS(mean±SD,month)	9.9±4.7	9.5±4.5	12.9±5.6	<0.001
No	18(6.5)	11(4.5)	7(21.9)	<0.001
Yes	261(93.5)	236(95.5)	25(78.1)	
OS(mean±SD,month)	24.5±5.2	24.2±5	26.9±6	0.006
No	207(74.2)	179(72.5)	28(87.5)	0.068
Yes	72(25.8)	68(27.5)	4(12.5)	

Table 2: Multivariate logistic regression for predicting PFS and OS.

Variable	PFS multivariable analysis		OS multivariable analysis	
	HR (95% CI)	p val	HR (95% CI)	p val
Age (>70 vs ≤70 years)	0.749(0.444-1.262)	0.336	0.515(0.244-1.087)	0.082
PSA(>66 vs ≤66 ng/ ml)	0.879(0.550-1.406)	0.592	0.792(0.401-1.567)	0.504
Histology(Other VS Adenocarcinoma)	1.836(0.541-6.239)	0.554	1.788(0.380-8.417)	0.462
M stage(M1b+M1c VS M10+M1a)	0.821(0.438-1.540)	0.453	1.133(0.410-3.129)	0.810
Gleason score(9-10 9-10VS 6-8)	1.035(0.623-1.721)	0.973	0.707(0.342-1.463)	0.350
Prior prostate RT and RP(no VS yes)	1.272(0.761-2.126)	0.806	1.022(0.474-2.203)	0.957
Prior therapy at mCRPC stage(Other VS Abiraterone)	1.181(0.679-2.054)	0.367	1.088(0.504-2.348)	0.830
Diabetes(yes VS no)	1.397(0.674-2.895)	0.733	0.467(0.143-1.526)	0.207
Metformin (yes VS no)	0.685(0.319-1.468)	0.220	1.616(0.449-5.815)	0.463
PTEN alteration (yes VS no)	0.718(0.398-1.296)	0.637	1.090(0.498-2.385)	0.829
BRCA alteration(yes VS no)	0.970(0.571-1.647)	0.622	0.804(0.351-1.842)	0.606
PTEN+/ Metformin+ (yes VS no)	3.242(1.013-10.379)	<0.001	10.646(1.032-109.835)	0.047

Table 3: Univariate and multivariate logistic regression for predicting PSA50

Variable	Univariate analysis		Multivariable analysis	
	HR (95% CI)	p val	HR (95% CI)	p val
Age (>70 vs ≤70 years)	0.794(0.407-1.546)	0.497	0.835(0.386-1.807)	0.647
PSA(>66 vs ≤66 ng/ ml)	1.789(0.912-3.508)	0.091	1.697(0.791-3.640)	0.174
Histology(Other VS Adenocarcinoma)	3.026(0.330-27.781)	0.328	6.273(0.458-85.891)	0.169
M stage(M1 VS M0)	0.470(0.182-1.209)	0.055	0.380(0.129-1.122)	0.023
Gleason score(9-10 9-10VS 6-8)	1.077(0.529-2.191)	0.838	0.788(0.384-1.784)	0.568
Prior prostate RT and RP(no VS yes)	0.835(0.423-1.648)	0.602	0.825(0.380-1.795)	0.628
Prior therapy at mCRPC stage(Other VS Abiraterone)	0.619(0.267-1.437)	0.264	0.437(0.166-1.150)	0.094
Diabetes(yes VS no)	0.862(0.426-1.746)	0.681	0.244(0074-0.502)	0.120
Metformin (yes VS no)	1.951(0.821-4.633)	0.005	5.413(1.466-19.985)	0.005
PTEN alteration (yes VS no)	1.727(0.866-3.445)	0.118	3.022(1.184-7.717)	0.026
BRCA alteration(yes VS no)	2.905(1.430-5.900)	<0.001	2.756(1.265-6.005)	<0.001
